# Long Non-coding RNA *LINC00114* Facilitates Colorectal Cancer Development Through EZH2/DNMT1-Induced miR-133b Suppression

**DOI:** 10.3389/fonc.2019.01383

**Published:** 2019-12-17

**Authors:** Lv Lv, Liang He, Shaohua Chen, Yaqun Yu, Guosong Che, Xuan Tao, Shengtao Wang, Zhiyuan Jian, Xuemei Zhang

**Affiliations:** ^1^Department of Emergency and Trauma Surgery, Affiliated Hospital of Guilin Medical University, Guilin, China; ^2^Department of Anesthesiology, Affiliated Hospital of Guilin Medical University, Guilin, China; ^3^Department of Breast and Thyroid Surgery, Affiliated Hospital of Guilin Medical University, Guilin, China; ^4^Department of Hepatobiliary and Pancreatic Surgery, Affiliated Hospital of Guilin Medical University, Guilin, China; ^5^Gastrointestinal Surgery Department, Affiliated Hospital of Guilin Medical University, Guilin, China; ^6^Department of Pathology, Affiliated Hospital of Guilin Medical University, Guilin, China

**Keywords:** *LINC00114*, EZH2, DNMT1, miR-133b, methylation, colorectal cancer

## Abstract

This study aimed to identify the roles of the long non-coding RNA *LINC00114* in colorectal cancer (CRC) development. The expression levels of *LINC00114* and miR-133b in CRC were determined by reverse transcription (RT)-polymerase chain reaction (PCR) and the functions of *LINC00114* in CRC were evaluated *in vitro* and *in vivo*. Methylation-specific PCR assay was performed to detect the miR-133b promoter methylation in CRC cells. Bioinformatics analysis, RNA immunoprecipitation, dual luciferase assay, RNA pull-down, co-immunoprecipitation (IP), and chromatin IP (ChIP) assays were used to elucidate whether *LINC00114* could recruit EZH2/DNMT1 and bind to the miR-133b promoter region, leading to dysregulated methylation and the depression of miR-133b. The expression levels of DNA methyltransferases (DNMTs), EZH2, and nucleoporin 214(NUP214) were analyzed by western blotting. Data showed that *LINC00114* was highly expressed, whereas miR-133b was downregulated in the CRC tissues and cells. *In vitro*, silencing *LINC00114* inhibited cell proliferation and impeded cell cycle at the G1/S phase by upregulating miR-133b. *In vivo, LINC00114* knockdown reduced tumor growth. Further analysis showed that the methylation in miR-133b promoter region was increased in the CRC and silencing *LINC00114* increased miR-133b expression through depressing methylation of its promoter region. ChIP-PCR experiments demonstrated that EZH2 and DNMT1 could bind to the miR-133b promoter region and it was abolished by *LINC00114* knockdown. sh-EZH2 reversed the overexpression of DNMTs and CRC cell cycle progression induced by the *LINC00114* upregulation. LINC00114 could regulate the NUP214 protein expression by sponging miR-133b. These results demonstrated that *LINC00114* suppressed miR-133b expression via EZH2/DNMT1-mediated methylation of its promoter region, indicating that *LINC00114* might be a potential novel target for CRC diagnosis and treatment.

## Introduction

Colorectal cancer (CRC) is the third leading cause of cancer-related deaths worldwide (1). Importantly, CRC incidence rates have been increasing rapidly in several countries that have been historically at low risk for CRC (2), and relative survival rates indicate poor 5-year CRC prognosis (1). Although recently developed medical techniques have improved diagnosis and treatment, unfortunately, CRC is usually detected at advanced stages, which significantly impedes its treatment (3). Therefore, elucidating early diagnostic markers of CRC and discovering the pathogenesis of CRC are critical.

Long non-coding RNAs (lncRNAs) are a class of nucleotides, approximately 200 bp in length with no protein-coding capacity. Accumulated evidences indicate that lncRNAs play vital roles in tumor growth and metastasis (4). lncRNAs are categorized into several types and exert their functions through different molecular mechanisms. Notably, lncRNAs were reported to be intimately related to the development and progression of CRC. The lncRNA MAPKAPK5 antisense RNA 1, which is upregulated in CRC, can promote the development of CRC by binding to p21 (5). Moreover, nuclear enriched abundant transcript 1, another lncRNA, facilitates the progression of CRC by binding to DEAD-box helicase 5 (DDX5) via activation of the Wnt/β-catenin signaling pathway (6). In addition, lncRNAs regulate the transcription of downstream molecules by regulating DNA methylation or hydroxymethylation as well as histone acetylation or methylation. Hypermethylation often occurs in the promoter region near the tumor suppressor genes transcription factor 21 (*TCF21*) and TCF21 antisense RNA inducing promoter demethylation (*TARID*), resulting in gene silencing. The LncRNA Chaer (cardiac hypertrophy associated epigenetic regulator) can directly bind to the catalytic domain of polycomb repressive complex 2 (PRC2) and interfere with PRC2 binding to target gene sites. This binding suppresses histone H3K27 methylation in the promoter region of cardiac hypertrophy-related genes, thereby affecting their expression (7). However, the roles of lncRNAs in CRC tumorigenesis remain to be elucidated.

miR-133b was previously identified as a tumor suppressor and was shown to inhibit cell proliferation and tumorigenesis in several cancers, such as prostate cancer (8), breast cancer (9), ovarian cancer (10), and CRC (11). Additionally, a variety of validated miR-133b targets including nuclear pore protein nucleoporin 214 (NUP214) (12), CXC chemokine receptor-4 (CXCR4) (13), and B cell lymphoma-2 (BCL-2) (14) are involved in mitosis, tumorigenesis, and apoptosis. Although these studies ascribe an antitumor role for miR-133b, no studies to date have investigated lncRNA-mediated epigenetic mechanisms that regulate miR-133b expression in CRC.

In the current study, we elucidated the mechanism underlying the modulation of miR-133b expression by *LINC00114*, which was determined by bioinformatics analysis. We determined whether *LINC00114* suppressed miR-133b expression via DNA methylation in CRC and demonstrated that *LINC00114* could directly inhibit CRC progression via miRNA sponging, providing a potential mechanism that might be utilized in CRC diagnosis and treatment.

## Materials and Methods

### Tissues and Cells

The human colon epithelial cell line NCM460 and the CRC cell lines HT-29, HCT116, SW620, and LoVo were purchased from the Cell Bank of Chinese Academy of Sciences (Shanghai). NCM460 cells were incubated in McCoy's 5a medium with 10% fetal bovine serum (FBS; Gibco, California, USA). The CRC cell lines were cultured in DMEM medium (Hyclone, Logan, UT, USA) containing 10% FBS. All cells were cultured with 5% CO_2_ at 37°C.

CRC specimens were obtained from Affiliated Hospital of Guilin Medical University, and adjacent normal tissues at least 3 cm away from the tumor border were isolated for analyses. Tissue samples were preserved in liquid nitrogen for transportation and stored at −70°C. The use of the specimens was approved by the Institutional Review Board of Affiliated Hospital of Guilin Medical University.

### *In situ* Hybridization

For *in situ* hybridization (ISH), CRC, and adjacent normal tissue specimens were fixed with 4% para-formaldehyde, dehydrated, and embedded in paraffin. The specimens were sliced into 4-μm-thick sections and mounted onto charged slides. After dewaxing and hydration, the sections were air-dried and immersed in distilled water containing 3% hydrogen peroxide, followed by immersion in pepsin solution for 30 min at 37°C. The sections were treated with hybridization buffer for 2 h at 37°C. Next, hybridization was performed by incubating the sections with the target DIG-labeled probe (AAGAAGCTGCTGAAGAACCCA) at 42°C overnight, followed by washing with 2 ×, 0.5 ×, and 0.2 × SSC solution, respectively. The sections were then blocked with biotinylated digoxin (Boster Biological Technology, Wuhan) for 1 h at 37°C and the streptavidin-biotin-peroxidase complex (Boster) for 20 min at 37°C. Hybridization signals were detected using DAB (3,3′-diaminobenzidine; P013IH, Auragene), and the sections were counterstained with hematoxylin. After dehydration, the sections were mounted with neutral gum and observed under the microscope.

### Transfection

*LINC00114* interfering plasmid (pGMLV-hU6-MCS-CMV-ZsGreen1-WPRE) and sequences are GCCGATTAAGGTCTGAGAAGT, GCCAACCACACAAGAAATAGG, and GCACATCATCATTGTGCTTCT), LINC00114 overexpression plasmid (pcDNA3.1) and enhancer of zeste 2 polycomb repressive complex 2 subunit (EZH2) interfering plasmid (sequences are GCTCCTCTAACCATGTTTACA, GCCAACCACACAAGAAATAGG, and GCTCCTCTAACCATGTTTACA) were obtained from Auragene (Changsha, China). For transfection experiments, cells were plated in six-well plates at a density of 4 × 10^5^ cells per well and transfected using Lipofectamine ®2000 (ThermoFisher Scientific, CA, USA), according to the manufacturer's protocol. After 48 h of incubation, the cells were collected for further analyses.

### MTT Assay

Cells were plated into 96-well plates at a density of 5,000 cells/well and were treat with or without miR-133b inhibitor 12 h later. After a another incubation for 24, 48, and 72 h, 10 μl MTT (3-(4,5-dimethylthiazol-2-yl)-2,5-diphenyltetrazolium bromide) solution (Sangon Biotech, China) was added to each well, the cells were cultured for 4 h at 37°C, and the medium was removed. Next, the cells were incubated with 150 μl dimethyl sulfoxide solution (MP Biomedicals, USA) for 10 min for cell lysis. The absorbance was measured at 570 nm using the Multiskan MK microplate reader (ThermoFisher Scientific, USA). All experiments were repeated three times.

### TUNEL Assay

For the terminal deoxynucleotidyl transferase dUTP nick-end labeling (TUNEL) assay, cells were fixed in 4% paraformaldehyde, incubated with permeabilization buffer containing Triton X-100 (Sigma), fluorescence-labeled reagent (45 μl) and terminal deoxynucleotidyl transferase (5 μl) (cat. #, 24529300; Sigma) for 1 h at 37°C. The nuclei were labeled with 4′,6-diamidino-2-phenylindole (Solarbio) for visualization. A fluorescence microscope system (Leica, Germany) was used to observe and capture images of the cells.

### Flow Cytometry

Forty-eight hours after transfection, the cells were collected, washed three times with cold phosphate-buffered saline (PBS) and fixed with 70% ethanol overnight. Next, the cells were stained with propidium iodide (PI; Keygentec, China) for 30 min at 4°C in the dark for cell cycle analysis. The cell cycle status was evaluated using a flow cytometer (Beckman Coulter, USA). Apoptosis of cells was assessed 48 h after transfection using an Annexin V-fluorescein isothiocyanate (FITC) apoptosis detection kit. Briefly, the cells were collected, washed twice with cold PBS, resuspended in 100 μL binding buffer, and incubated at room temperature in the dark for 30 min.

### Animals

The right flanks of BALB/c nude mice (aged 4–5 weeks) were subcutaneously injected with 1 × 10^6^ stable expression of LINC00114 -sh HCT116 cells (*n* = 4 per group). After 4 weeks, the animals were sacrificed and the weight and volume of tumors were measured. Next, the tumor was cut into two pieces. One piece was fixed with 4% paraformaldehyde and sliced into 5-μm-thick sections for immunohistochemistry assay. The other was stored in liquid nitrogen. All animal experiments were approved by the Animal Care Committee of Affiliated Hospital of Guilin Medical University. Immunohistochemistry Staining was performed using 5-μm-thick sections that were blocked with 10% FBS. After washing with PBS, the sections were incubated in TBST containing Ki67 (3174S, Cell Signaling Technologies, Danvers, MA, USA) overnight at 4°C. Next, the sections were washed with PBS and incubated with the secondary antibody for 30 min, followed by incubation with DAB and hematoxylin for 30 s to visualize the signal.

### Reverse Transcription-Polymerase Chain Reaction

Total RNA was extracted from tissues and cells using TRIZOL® (Invitrogen). The PrimeScript RT reagent kit (Takara, Dalian, China) was used for reverse transcription, and SYBR® Premix DimerEraser (Takara) and StepOnePlus real-time polymerase chain reaction (PCR) system (Applied Biosystems, Foster City, CA, USA) were utilized for RT-PCR. Primer sequences (Generay Biotechnology, Shanghai) are presented as follows: *LINC00114* (forward: TTGTGGCATCATAGCAGT, reverse: CTAAAGCATATTTGGGTGA), NUP214(forward: CGAGCGGGAGATGAAGGATTT, reverse: ACCAGCGAAGACCAGACCATA). TaqMan® 2 × universal master mix (Applied Biosystems) was used for quantitative PCR. Glyceraldehyde 3-phosphate dehydrogenase (GAPDH) served as the endogenous control. The relative expression of targets was calculated using the 2^−ΔΔCt^ method (15).

### Western Blotting

Cells were lysed using RIPA lysis buffer (Auragene) with a protease inhibitor cocktail (Roche, USA) and phenylmethylsulfonyl fluoride (Auragene). Equal amounts (25 μg) of protein were loaded on 10–12% sodium dodecyl sulfate-polyacrylamide gels, and the separated proteins were transferred to Immobilon-P polyvinylidene difluoride membranes (Millipore, USA). The membranes were blocked in TBST containing 3% BSA with gentle shaking for 90 min at room temperature and incubated with primary antibodies overnight at 4°C. After washing with TBST, the membranes were incubated with specific secondary antibodies 1 h at room temperature. The following primary antibodies from Abcam (Massachusetts, USA) were used: β-actin antibody (ab8226, 1:5,000), EZH2 (ab191250, 1:1,000), DNA methyltransferase 1 (DNMT1, ab92314, 1:1,000), H3K27me3 (ab6147, 1:500), DNMT3B (ab2851, 1:200), and NUP214 (ab70497, 1:2,000).

### RNA Immunoprecipitation

RNA immunoprecipitation (RIP) experiments were performed using EZMagna RIP kit (Millipore, Billerica, MA, USA), according to the manufacturer's instructions. Briefly, HCT116 and LoVo cells were lysed using the RIP lysis buffer, and the lysates were incubated with magnetic beads containing specific antibodies (Ago, EZH2) or control IgG (Millipore). The antibody against EZH2 (Abcam) was used at a 1:200 dilution. Next, the beads were washed and incubated with Proteinase K for protein removal. Finally, RT-qPCR assay was used to measure the RNAs.

### RNA Pull-Down

Briefly, T7 RNA polymerase (Promega, Madison, WI, USA) and biotinylated RNA-tagged mixtures (Roche, Basel, Switzerland) were used to label the transcriptional *LINC00114 in vitro*. Biotinylated *LINC00114* was then mixed and incubated with cell lysates. Streptavidin magnetic beads (Life Technologies) were added to each binding reaction and further incubated at room temperature for 1 h. Beads were then washed briefly three times and boiled in SDS buffer, and the retrieved protein was detected by standard western blotting.

### Chromatin Immunoprecipitation

For chromatin immunoprecipitation (ChIP), the Magna ChIP Kit (Millipore, Bedford, MA) was used in accordance with the manufacturer's instructions. Briefly, CRC cells were treated with 4% formaldehyde, and chromatin fragments with sizes ranging from 200 to 300 bp were generated by sonicating the cell lysates. Next, chromatin was immunoprecipitated with anti-EZH2, anti-DNMT1, anti-DNMT3B, and anti-He3mek27 antibodies, all from Abcam. IgG was used as control. RT-PCR was performed to recover and measure the precipitated chromatin DNA. The forward primer sequences are TCAATGCTATCCCCTTGTTAAACC and the reverse are ACCAA TACCCATGAACAACG. The forward primer sequences of negative control (around −7,000) are GCCAAGTGACACACCACATC and the reverse GCATAGGGCAAGGGCATCTA. The amount of immunoprecipitated DNA was calculated and normalized to the amount of input DNA.

### Luciferase Reporter Assay

The predicted miR-133b combining site of LINC00114 was mutated and sequences were generated by chemical synthesis. The Wt-LINC0014 and Mut-LINC0014 overexpression constructs were provided by genepharma (Suzhou, China), with a psi-CHECK2 vector. HCT116 and LoVo cells were maintained and co-transfected with miR-133 mimics, inhibitors, or negative control (NC), respectively, using the Cellfectin®II reagent. After incubation for 48 h, the firefly luciferase activity in cells was determined using the dual luciferase reporter assay system (E1910; Promega).

### Methylation-Specific PCR Assay

For the methylation-specific PCR (MSP) assay, genomic DNA was processed with EZ DNA Methylation-Gold™ kit (Zymo Research, USA). PCR was performed with HotStar Taq polymerase (Qiagen, Germany) using the following conditions: initial incubation of 94°C for 4 min; 35 cycles of 94°C for 30 s, 62°C for 30 s, and 72°C for 30 s; and one cycle of 72°C for 5 min. PCR products were electrophoresed in 3% agarose gels and visualized by ultraviolet illumination. The miR-133b MSP primer sequences were as follows: forward, 3′-TTTATTTAAAATATAAAAATA GCGG-5′; reverse, 3′-TCACCCAAACTAAAATACAAT AACG-5′; unmethylated miR-133b forward, 3′-TTTTTA TTTAAAATATAAAAATAGTGG-5′; unmethylated miR-133b reverse, 3′-ACC CAAACTAAAATACAATAACACT-5′.

### Statistical Analysis

Data were presented as means ± standard error. All experiments were repeated at least three times. All data was analyzed using GraphPad Prism 7.0 (CA, USA). Multiple comparisons were analyzed using one-way analysis of variance, and differences between groups were compared using Dunnett's multiple comparisons test. *P* < 0.05 was considered statistically significant.

## Results

### Expression Levels of *LINC00114* and mIR-133b in CRC Tissues and Cell Lines

**Figure 1:**
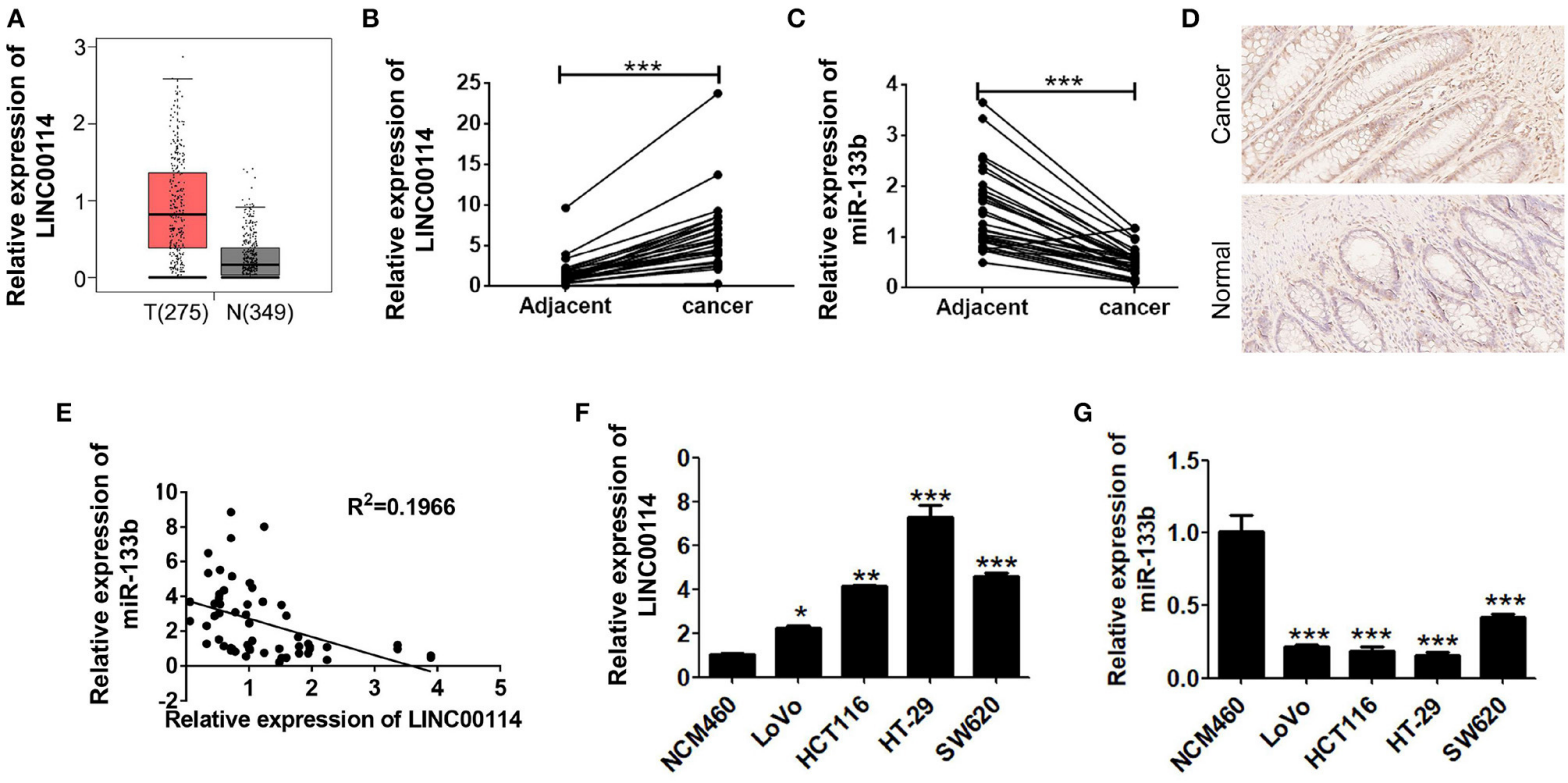
*LINC00114* is upregulated in CRC. **(A)** Highly expressed *LINC00114* is predicted in the GEPIA database. **(B)** *LINC00114* is upregulated in the CRC samples compared to the adjacent normal tissue samples, determined via RT-PCR assay. **(C)** RT-PCR result shows that expression of miR-133b expression is reduced in the CRC samples. **(D)** *LINC00114* expression is higher in the CRC as measured by *in situ* hybridization (200X). **(E)** There is an inverse correlation between *LINC00114* and miR-133b expression in the CRC tissue samples. **(F,G)** The expression levels of *LINC00114* and miR-133b in the CRC cells are determined by RT-PCR. *P < 0.05; **P < 0.01; ***P < 0.001.

We previously demonstrated that miR-133b expression was reduced in CRC, although the upstream regulatory mechanism remained unclear (11). Therefore, we first analyzed the TCGA database to see if there was any lncRNAs regulating miR-133b as a upstream regulator and found that there were 1,046 lncRNAs upregulated in CRC. We next investigated the potential targets of miR-133b using the miRanda software and *LINC00114* was identified as a potential target of miR-133b. The relatively high expression level of *LINC00114* in CRC was also verified in the GEPIA database (http://gepia.cancer-pku.cn/) (Figure 1A). To verify whether *LINC00114* and miR-133b were both dysregulation in CRC, we next performed RT-PCR and ISH assays and found that *LINC00114* was upregulated while miR-133b was downregulated in the CRC specimens compared with the adjacent tissues (Figures 1B–D), suggesting a negative correlation between *LINC00114* and miR-133b (Figure 1E). Additionally, the expression trend of *LINC00114* and miR-133b in CRC cells was same with it in tissues (Figures 1F,G).

### *LINC00114* Regulates CRC Progression Through Inhibiting miR-133b Expression *in vitro* and *in vivo*

**Figure 2:**
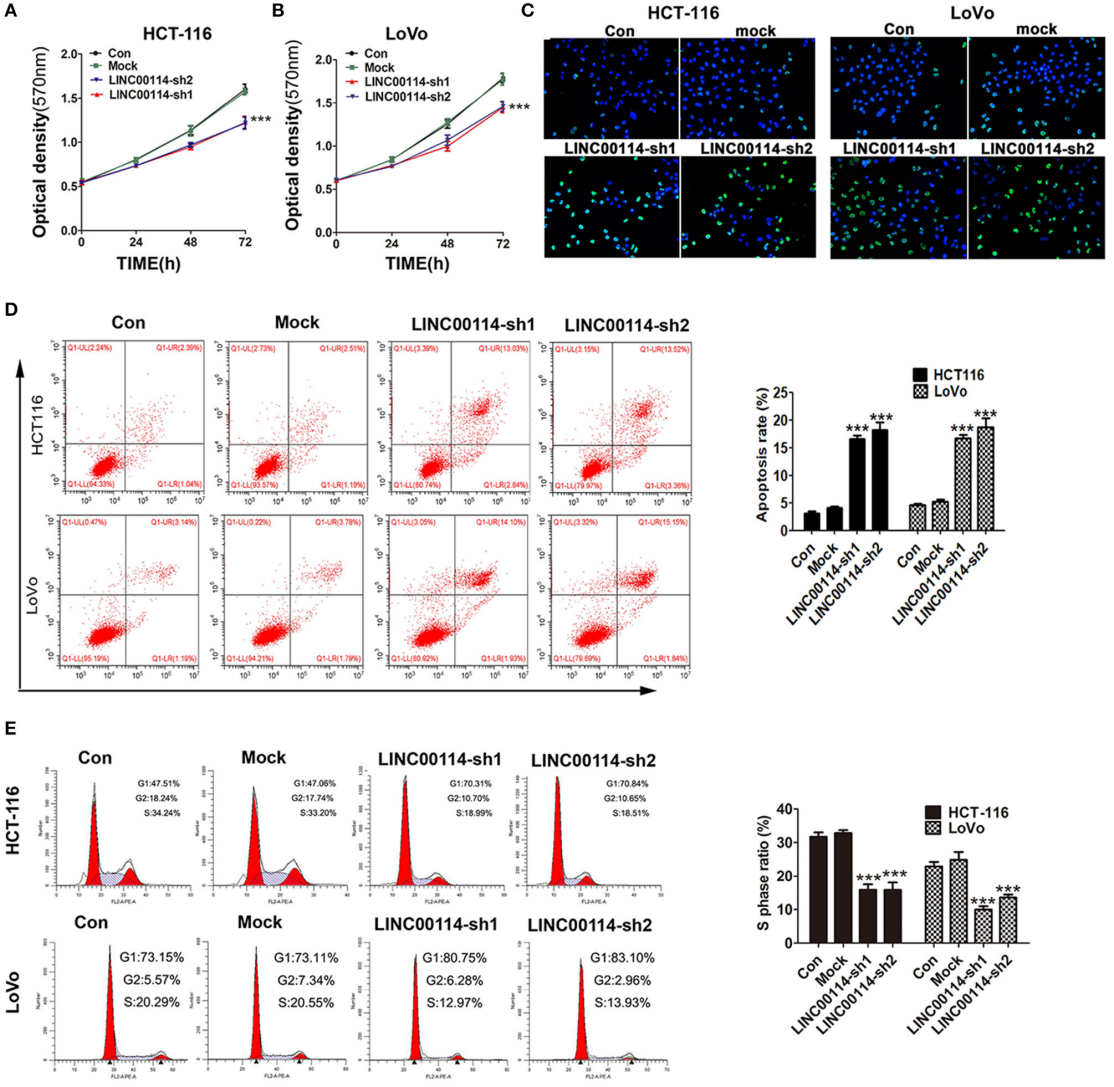
*LINC00114* interference facilitates apoptosis and represses cell cycle progression *in vitro*. **(A–C)** sh-LINC00114 inhibits cells proliferation. **(D)** Apoptosis of CRC cells transfected with the sh-LINC00114 or the control plasmid for 48 h, analyzed by flow cytometry. **(E)** Cell cycle analysis of HCT116 and LoVo cells transfected with the *LINC00114* or the control plasmid for 48 h using flow cytometry. ***P < 0.001. Con, the group only treated with PBS; Mock, the cells transfected with the empty vector plasmid.

**Figure 3:**
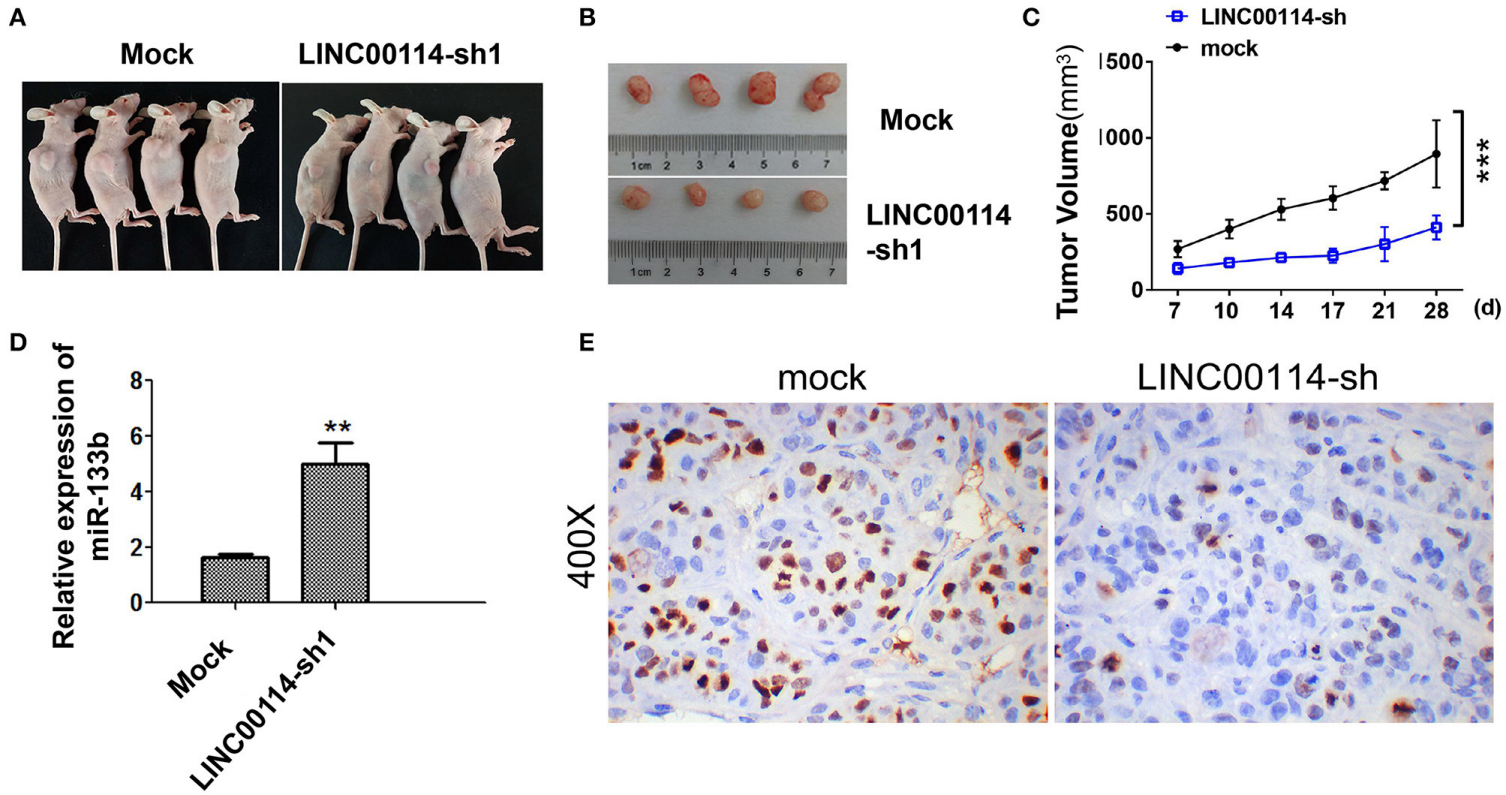
*LINC00114* interference inhibits the development of CRC *in vivo*. **(A)** Tumor formation in nude mice. **(B)** Tumor body in nude mice. **(C)** Tumor growth curve. **(D)** Expression of miR-133b in tumor tissues. **(E)** Ki67 expression determined by immunohistochemistry. **P < 0.01; ***P < 0.001.

To determine whether *LINC00114* promoted the progression of CRC, we transfected sh-LINC00114 plasmids (LINC00114-sh1 and LINC00114-sh2) into HCT116 and LoVo cells (Figure S1). The results showed that *LINC00114* knockdown inhibited the proliferation of these cells (Figures 2A,B) and the result was further confirmed by flow cytometry and TUNEL assays (Figures 2C,D). What's more, silencing *LINC00114* disrupted the transition of G1/S in HCT116 and LoVo cells (Figure 2E). We also performed a nude mouse xenograft model to explore the effects of *LINC00114 in vivo* and found that, consistent with the findings above, *LINC00114* suppression significantly reduced the tumor growth, compared with the control group (Figures 3A–C). RT-PCR and immunohistochemistry analysis revealed that the expression of miR-133b was promoted and the signal of Ki67 in the tumors of *LINC00114* suppression group was weaker than that in the control group (Figures 3D,E). All these data firmly demonstrated that *LINC00114* played vital roles in the growth of CRC *in vivo* and *in vitro*.

### *LINC00114* Regulates the Methylation of miR-133b Promoter Region

**Figure 4:**
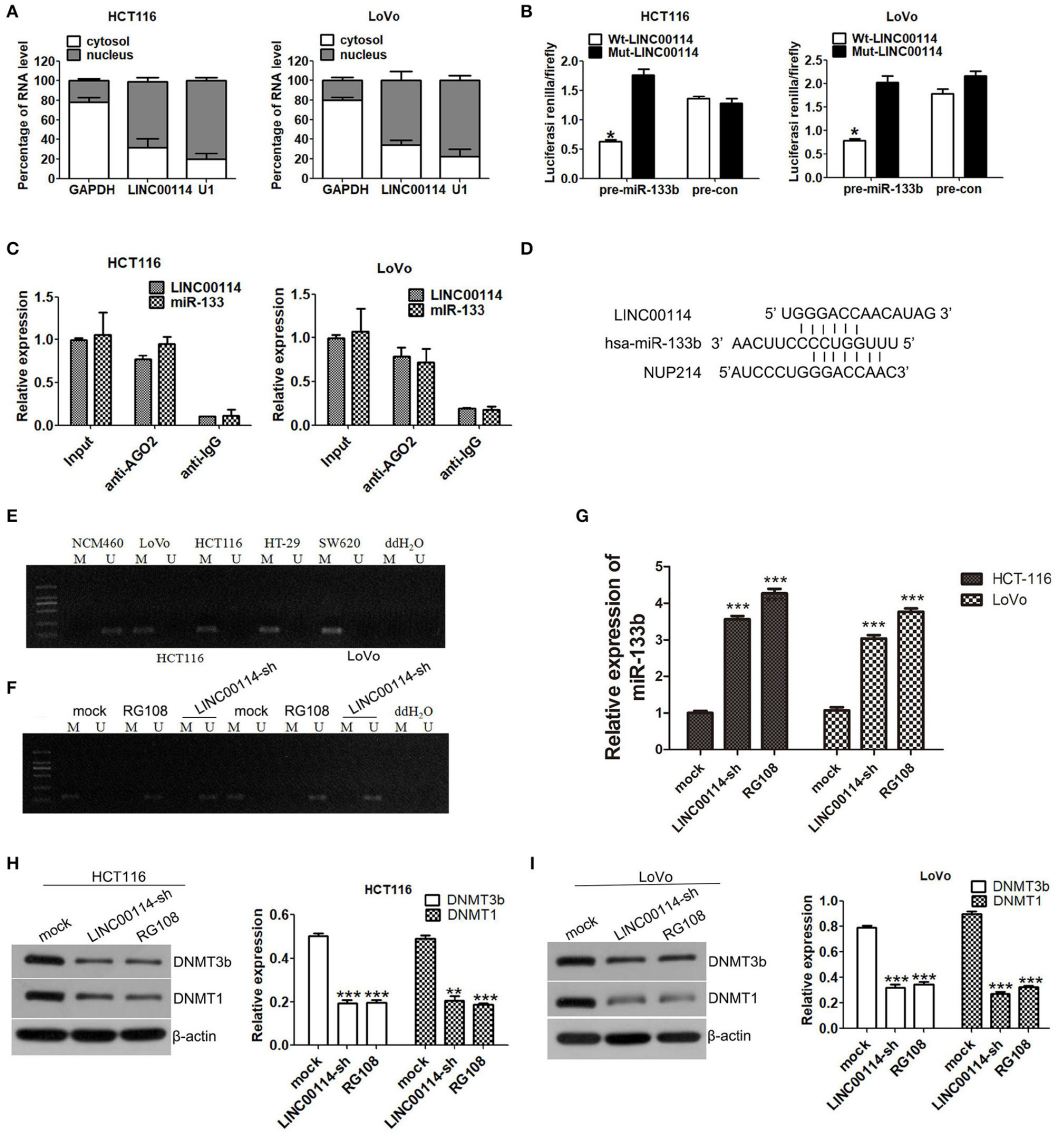
*LINC00114* regulates DNA methylation of the *miR-133b* promoter region. **(A)** Expression levels of *LINC00114* in the nucleus and cytoplasm of HCT116 and LoVo cells. **(B)** Luciferase activity of cells cotransfected with miR-133b overexpression and control plasmids after *LINC00114* overexpression or mutation. **(C)** RIP assay was used to analyse the binding of *LINC00114* to miR-133b. **(D)** The schematic diagram showed the binding site of miR-133b with *LINC00114* and NUP214 mRNA. **(E)** Methylation detection of miR-133b promoter region in cells. **(F)** *LINC00114* interference and RG108 treatment reduced the methylation levels of miR-133b promoter region in HCT116 and LoVo cells. **(G)** miR-133b levels after *LINC00114* interference and RG108 treatment reduced the expression of miR-133b in cells. **(H,I)** Western blotting result showed that DNMT1 and DNMT3B were reduced in *LINC00114-*silenced or RG108-treated cells. *P < 0.05; **P < 0.01; ***P < 0.001. M, methylation. U, non-methylation.

To explore the mechanism underlying *LINC00114*-mediated CRC cell proliferation, we then investigated the localization of *LINC00114* (http://www.rna-society.org/rnalocate/) and found that *LINC00114* was localized in both the cytoplasm and the nucleus (60 and 40%, respectively) (Figure 1D). These were further confirmed by RT-PCR assay (Figure 4A). We next used the dual luciferase reporter and RIP assays to verify that miR-133b and *LINC00114* could bind to each other in a direct or indirect manner (Figures 4B–D). Together, these data identified that *LINC00114* could bind to miR-133b. Previous studies demonstrated that the methylation level of miR-133b promoter region was high in CRC (11). We further confirmed these using MSP assay, as shown in Figure 4E. The miR-133b methylation level was low in NCM460 while high in CRC cells (LoVo, HCT116, HT-29, and SW620). Considering that *LINC00114* depletion increases the expression of miR-133b (Figure 3D), we were urged to explore if *LINC00114* regulates the expression of miR-133b by regulating methylation levels in its promoter region. To this end, we transfected LoVo and HCT116 cells with LINC00114-sh1 or treated with RG108, a DNA methyltransferase inhibitor, and found that *LINC00114* knockdown and RG108 treatment reduced the methylation level of the miR-133b promoter region both (Figure 4F), as we expected. The treatments also increased the expression of miR-133b in CRC cells (Figure 4G), indicating that *LINC00114* might regulate the methylation level in miR-133b promoter region.

It is known that DNMT3A and DNMT3B enzymes are important for the DNA methylation (16, 17), and DNMT1 also is a designated maintenance DNA methyltransferase, in a replication-dependent manner DNMTs (18, 19). To investigate the roles of these DNMTs in the *LINC00114*-regulated change of miR-133b promoter methylation, we performed western blotting assays and found that the expression of DNMT1 and DNMT3B were decreased in the cells with *LINC00114* silenced or RG108 treatment (Figures 4H,I), suggesting that *LINC00114* might regulate the methylation of the miR-133b promoter through methyltransferases.

### *LINC00114* Regulates Methylation Level of miR-133b Promoter by Binding to EZH2

**Figure 5:**
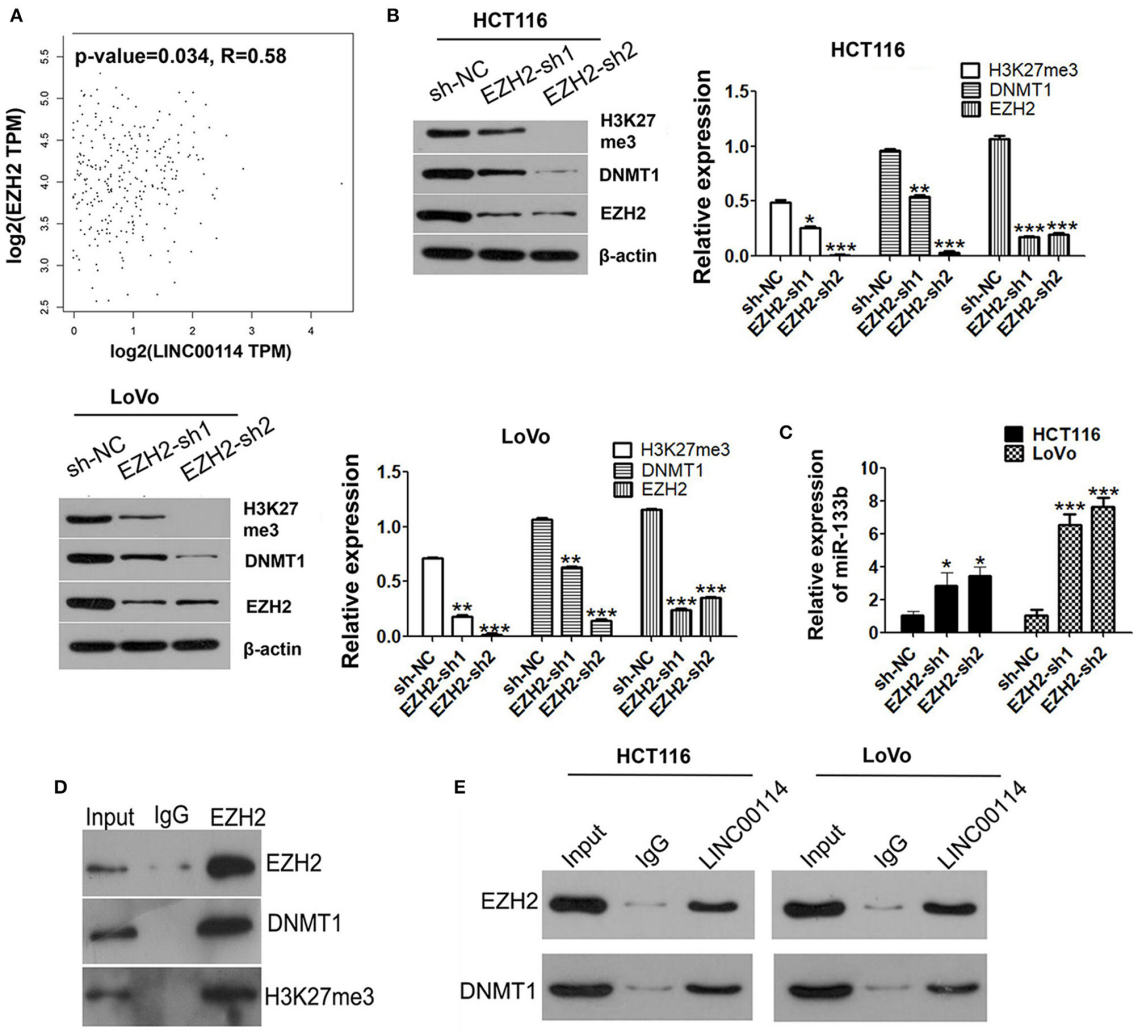
*LINC00114* binds to EZH2 protein. **(A)** Correlation analysis of *LINC00114* and EZH2 using the GEPIA database, also see it in method section. **(B)** EZH2 interference plasmids were transfected into HCT116 and LoVo cells and then H3K27me3, DNMT1, and EZH2 were detected using western blotting assay. **(C)** miR-133b levels were detected by RT-PCR in cells transfected with sh-EZH2. **(D)** Co-IP analysis showed that EZH2 could bind to DNMT1 and H3K27me3. **(E)** RNA pull-down assay was performed to detect the binding of *LINC00114* with EZH2 and DNMT1. *P < 0.05; **P < 0.01; ***P < 0.001.

Several studies had been reported that the function of some lncRNAs was EZH2-depended and participated in tumor progression by recruiting PRC2 (20, 21). Therefore, we first explored whether *LINC00114* could bind to DNA methyltransferase using online RNA-protein interaction prediction software (http://pridb.gdcb.iastate.edu/RPISeq/). As expected, it showed that *LINC00114* might bind to EZH2 and DNMT1 (with RF and SVM scores of >0.5), which did bind to miR-133b (Table S1). We also confirmed that *LINC00114* expression was positively correlated with EZH2 using GEPIA (http://gepia.cancer-pku.cn/index.html) (Figure 5A). Further, we found that the expression levels of EZH2, DNMT1, and H3K27me3 were reduced when EZH2 knocked down (Figure 5B). Also, EZH2 depression promoted the expression of miR-133b (Figure 5C) and EZH2 indeed binds with DNMT1 and H3K27me3 as shown in the result of Co-IP assay (Figure 5D). Moreover, the RNA pull-down assay revealed that biotinylated *LINC00114* could enrich the EZH2 and DNMT1 proteins in cells (Figure 5E) and *LINC00114* was enriched in the RNA-protein complexes immunoprecipitated by EHZ2 and DNMT1. These results confirmed the interplay between *LINC00114*, EZH2, and DNMT1.

**Figure 6:**
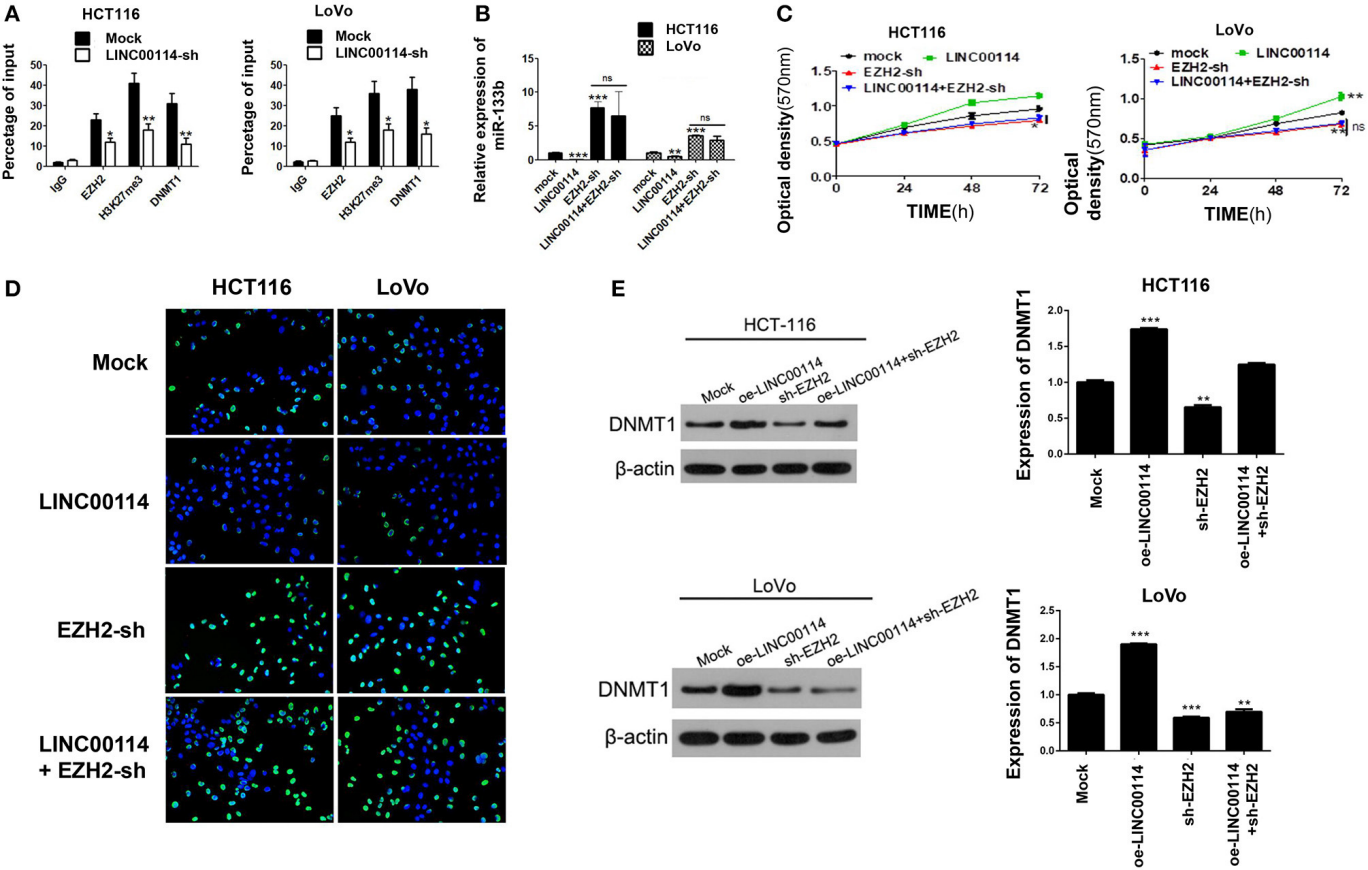
LINC00114 promotes the methylation of miR-133b promoter region by recruiting EZH2 and DNMT1. **(A)** *LINC00114* affects EZH2, DNMT1, and H3K27me3 binding with the miR-133b promoter region in CRC cells, as detected by ChIP assay. **(B)** EZH2-sh reversed the LINC00114-induced inhibition of miR-133b in cells. **(C,D)** MTT and TUNEL assays result showed that EZH2-sh reversed the LINC00114-incduced proliferation promotion of cells. **(E)** EZH2-sh reversed the upregulation of DNMT1 induced by *LINC00114* overexpression. oe, overexpression. *P < 0.05; **P < 0.01; ***P < 0.001.

To investigate whether DNMTs and EZH2 were involved in the *LINC00114 -*mediated methylation of miR-133b promoter, we performed ChIP assay and it showed that the knockdown of *LINC00114* reduced the binding of the DNMTs and EZH2 to the miR-133b promoter region (Figure 6A). This result indicated that *LINC00114* altered the EZH2-mediated H3K27me3 trimethylation, thereby leading to the miR-133b repression in hypermethylation-depended manner. In addition, sh-EZH2 neutralized the miR-133b depression-mediated by *LINC00114* overexpression (Figure 6B). The TUNEL and MTT assays revealed that *LINC00114* overexpression failed to promote the proliferation of CRC cells, owing to EZH2 depression (Figures 6C,D). Rescue experiments demonstrated that sh-EZH2 attenuated the effect of *LINC00114* overexpression on DNMT1 expression (Figure 6E). These results indicated that *LINC00114* promoted miR-133b promoter methylation by inducing the EZH2/DNMT1 binding to the miR-133b promoter region, thereby facilitating the development of CRC.

### *LINC00114* Regulates CRC Cell Proliferation by Sponging miR-133b

**Figure 7:**
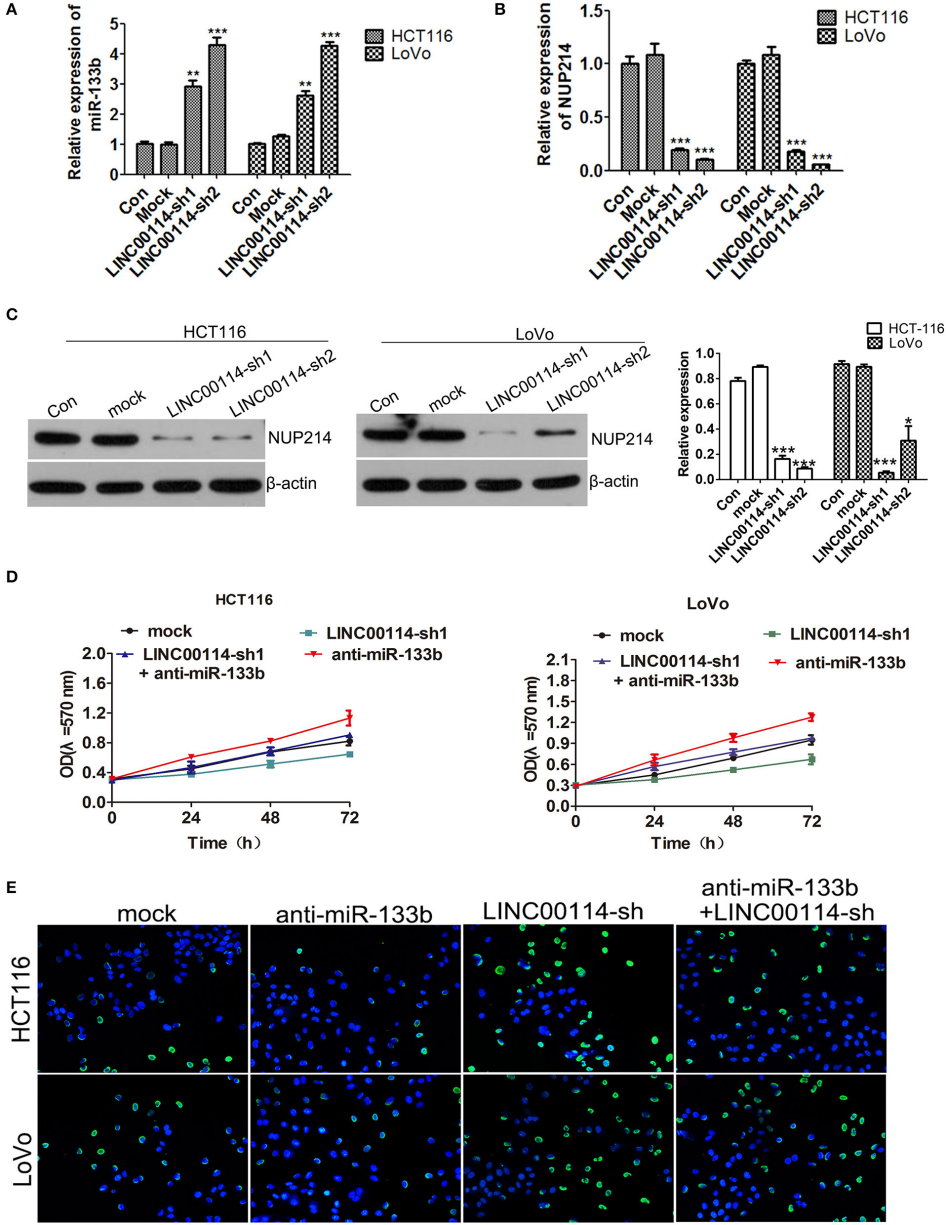
*LINC00114* regulates cell proliferation via sponging miR-133b. **(A)** Expression of miR-133b detection in *LINC00114* silenced cells. **(B,C)** The expression of NUP214 was determined by RT-PCR and western blotting, respectively. **(D,E)** Inhibition of miR-133b maintained proliferation promotion of cells in *LINC00114* silenced cells, as shown in MTT and TUNEL assays, respectively. *P < 0.05; **P < 0.01; ***P < 0.001.

Previous study reported that miR-133b could target NUP214, a member of the massive nuclear pore complex, illustrating that the miR-133b-mediated NUP214 downregulation arrested cells in the early mitotic phase and led to aberrant chromosome segregation and cell death (12). Thus, we assessed whether *LINC00114* regulated cell cycle progression through NUP214. We next found that both NUP214 mRNA and *LINC00114* shared the binding site for miR-133b (Figure 4D). Furthermore, *LINC00114* knockdown facilitated miR-133b expression while depressed NUP214 expression in cells (Figures 7A–C). In addition, the assays revealed that miR-133b silencing promoted the CRC cell growth and that sh-*LINC00114* neutralized the proliferation promotion mediated by miR-133b inhibitor (Figures 7D,E), indicating that *LINC00114* regulated cell proliferation via sponging miR-133b.

## Discussion

Although radiotherapy chemotherapy and primary treatment approaches for CRC can prolong life expectancy and reduce patient burden, the CRC prognosis remains poor (22, 23). LncRNAs were recently demonstrated to play vital roles as major regulators of gene expression and cancer cell proliferation (24) in multiple cancers. Several lncRNAs have been identified as regulators in CRC cell proliferation and metastasis (25). In current study, we focused on a cancer-related lncRNA, *LINC00114*, which we found to be upregulated in CRC. Moreover, we demonstrated that *LINC00114* negatively regulated miR-133b expression via DNA methylation, suggesting that *LINC00114* contributed to the CRC development as an oncogene. We further found that *LINC00114* served as an miR-133b sponge to regulate NUP214 expression, thus participating in the progression of CRC.

On one hand, lncRNAs can competitively bind to miRNAs, regulating the proliferation and metastasis of cancer cells (26). Gastric cancer associated transcript 3, an lncRNA, is upregulated in colon cancer and it combines with miR-3127 via ELAV like RNA binding protein 1 to promote CRC (26). Moreover, nuclear enriched abundant transcript 1 is another lncRNA that can compete with sirtuin 1 to bind miR-34a and regulate the Wnt/β-catenin signaling pathway and promote the development of CRC (27). In the current study, we identified that knockdown of *LINC00114* significantly increased the expression of miR-133b, and *LINC00114* was inversely correlated with miR-133b in CRC. Numerous studies demonstrate that miR-133b plays an important role in CRC progression by inhibiting cell proliferation and metastasis (11, 12, 28). Our results showed that the knockdown of *LINC00114* inhibited CRC cell proliferation by negatively regulating miR-133b expression, indicating that *LINC00114* might promote the development of CRC by suppressing miR-133b.

Conversely, lncRNAs can regulate gene expression through various mechanisms, such as chromatin modification and pre- and post-transcriptional regulation. Growing evidences indicate that some lncRNAs regulate the expression of downstream target genes by DNA methylation (29, 30). In this study, we showed that silencing *LINC00114* decreased DNA methylation in the *miR-133b* promoter region. Previous study reported that lncRNAs were associated with several RNA-binding proteins including EZH2, SUZ12, LSD1, staufen double-stranded RNA binding protein 1, and DNMT1 (31, 32). EZH2 is a component of the methyltransferase in PRC2 and regulated by lncRNAs at the transcriptional level. EZH2 induces H3K27 methylation; it also induces LSD1 to regulate H3K4 demethylation (33). EZH2 usually mediates the methylation of the second lysine of histone H3 and is associated with tumor progression, angiogenesis, inflammation (34). Moreover, EZH2 may also recruit DNMT1 to the promoter region of its target genes. DNMTs and methyl-CpG binding protein 2 were reported to play an important role in methylation modification (35–37). These studies raise the possibility that lncRNAs may have a primary role in recruiting polycomb proteins to their target genes (32, 36). In our study, we found that *LINC00114*, together with EZH2 and DNMT1, played a direct role by online RIP-seq data. We hypothesized that *LINC00114* recruited EZH2 and DNMT1 to the *miR-133b* promoter region for its hypermethylation and repression of miR-133b expression. To validate the relationship among EZH2, *LINC00114*, and DNMTs, we performed an RNA pull-down assay and confirmed that EZH2 and DNMT1 were binding partners of *LINC00114*. RT-PCR analysis confirmed that knockdown of *LINC00114* affected the expression of DNMTs and miR-133b. In agreement with previous studies which reported that EZH2 could regulate DNA methylation through DNMTs (38), we determined that silencing EZH2 resulted in downregulation of DNMT1 and upregulation of miR-133b expression. Furthermore, knockdown of EZH2 reversed the *LINC00114*-induced DNMT1 upregulation and cell cycle progression. Additionally, knockdown of *LINC00114* reduced the containing of EZH2 and DNMT1 that bind to the promoter region of miR-133b. These results indicated that *LINC00114* might regulate the expression of miR-133b by binding to EZH2 and DNMT1.

It was reported that miR-133b impacted the development of CRC by regulating NUP214. Nucleopore is essential for many species to enter the nucleus, and NUP214 regulates mitosis and promotes carcinogenesis (39). NUP214 is involved in impeding mitotic progression and is a reported target of miR-133b; it is also highly expressed in HCT116 cells (12). In the current study, we demonstrated that *LINC00114* could regulate NUP214 protein expression by sponging miR-133b. In addition, miR-133b and NUP214 act as key factors in the pathogenesis of CRC. These findings also suggest that *LINC00114* affects the development of CRC by competitively binding to miR-133b through NUP214.

In conclusion, the current study demonstrated that *LINC00114* expression was increased in CRC, which might represent an unfavorable prognostic factor for CRC. In addition, *LINC00114* depletion inhibited cancer cell proliferation both *in vitro* and *in vivo*. Moreover, the oncogenic effects of *LINC00114* were facilitated by its binding to EZH2 and DNMT1 and consequent induction of miR-133b promoter methylation which led to the downregulation of miR-133b. Our results also revealed that *LINC00114* served as a molecular sponge for miR-133b, reversing its ability to repress NUP214 protein. Overall, these findings reveal novel therapeutic targets that should be considered for CRC.

## Data Availability Statement

Publicly available datasets were analyzed in this study. This data can be found here: http://gepia.cancer-pku.cn/.

## Ethics Statement

The studies involving human participants were reviewed and approved by The Ethics Committee of the Guilin Medical University. The patients/participants provided their written informed consent to participate in this study. The animal study was reviewed and approved by The Ethics Committee of the Guilin Medical University. Written informed consent was obtained from the individual(s) for the publication of any potentially identifiable images or data included in this article.

## Author Contributions

ZJ and XZ designed the experiments. LL and LH performed the most of the experiments. SC and YY analyzed the data. GC and XT wrote the paper. SW revised the paper.

### Conflict of Interest

The authors declare that the research was conducted in the absence of any commercial or financial relationships that could be construed as a potential conflict of interest.

## Supplementary Material

Figure S1 **(A,B)** Verification of the efficiency of *LINC00114* interference plasmid in HCT116 and LoVo cells. ****P* < 0.001.

Table S1 Prediction of the binding ability of *LINC00114* and mir-133b to genes.
