# Supplementary figures and images for: Long Non-coding RNA LINC00114 Facilitates Colorectal Cancer Development Through EZH2/DNMT1-Induced miR-133b Suppression
Source: Front Oncol. 2019 Dec 17;9:1383. doi: 10.3389/fonc.2019.01383 (PMC6928983; doi:10.3389/fonc.2019.01383)

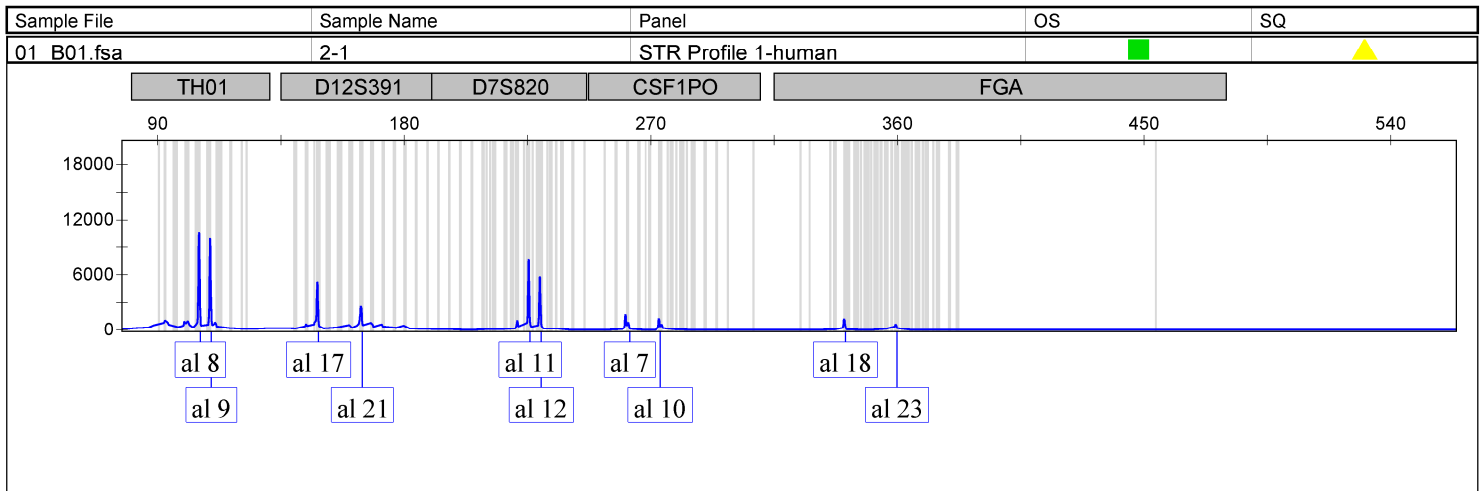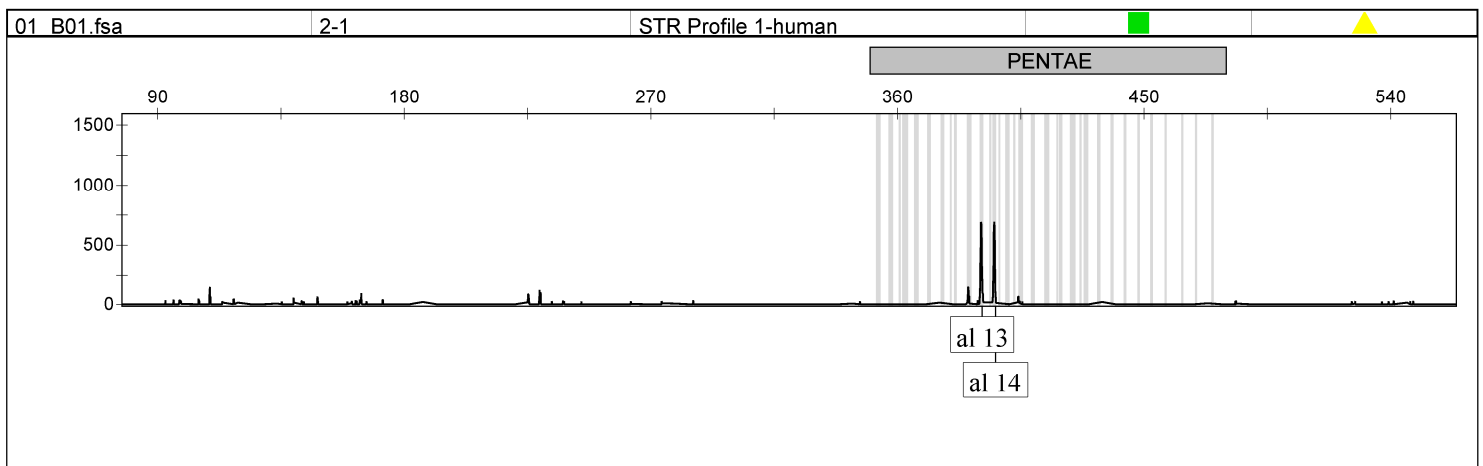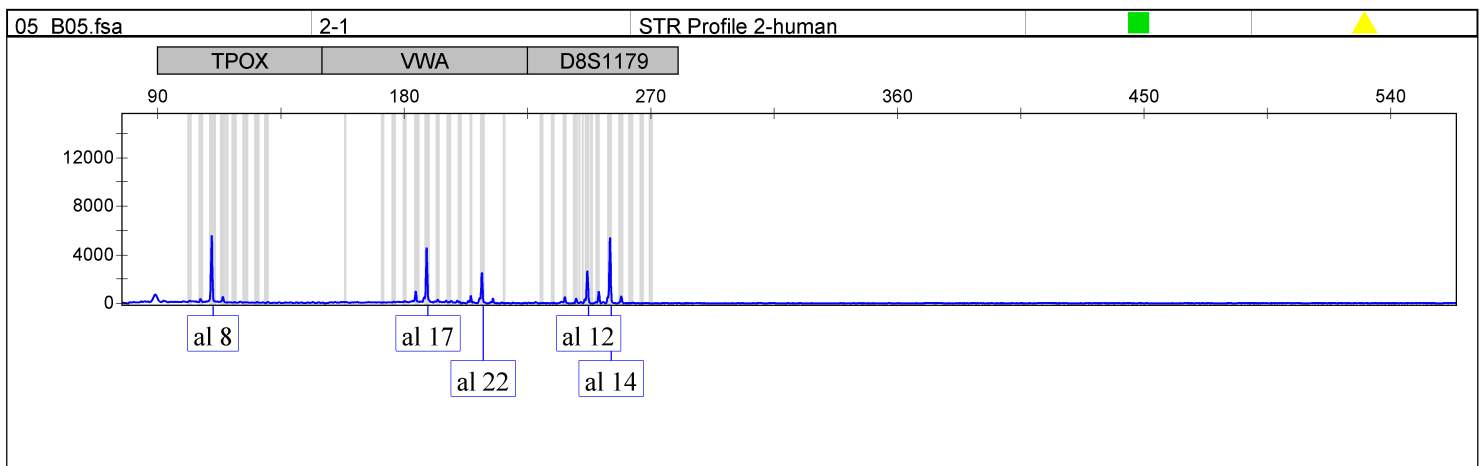

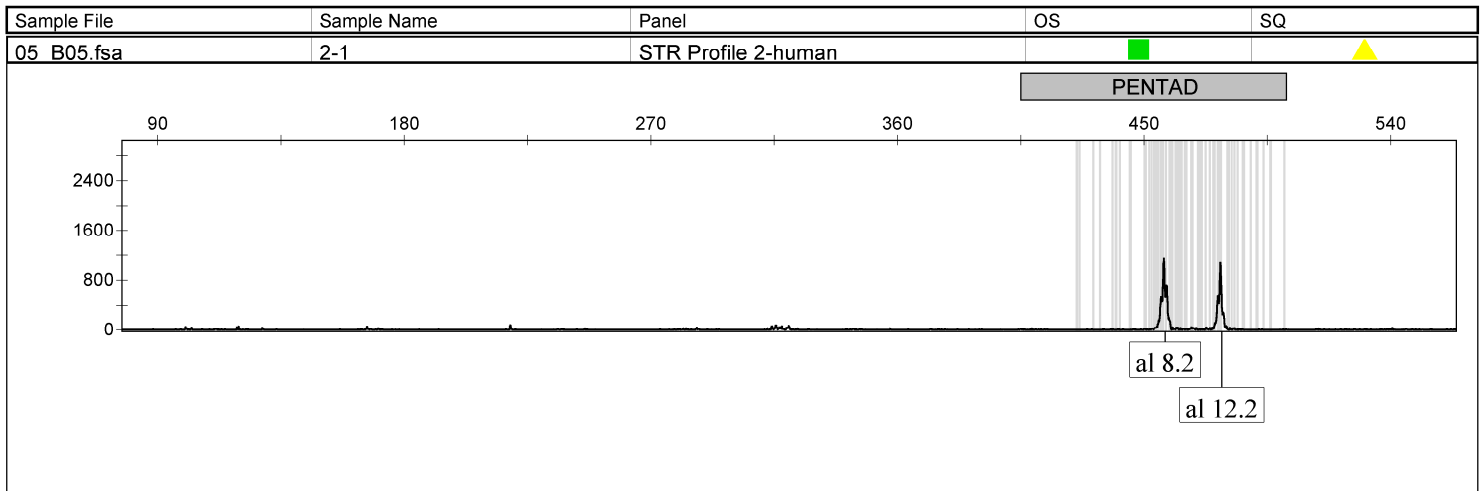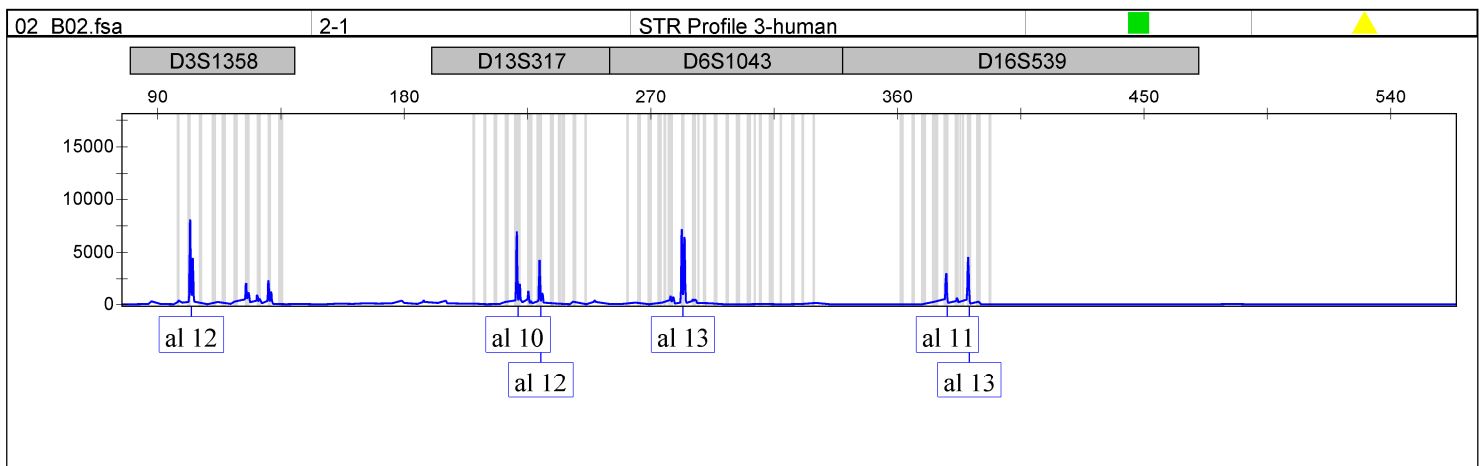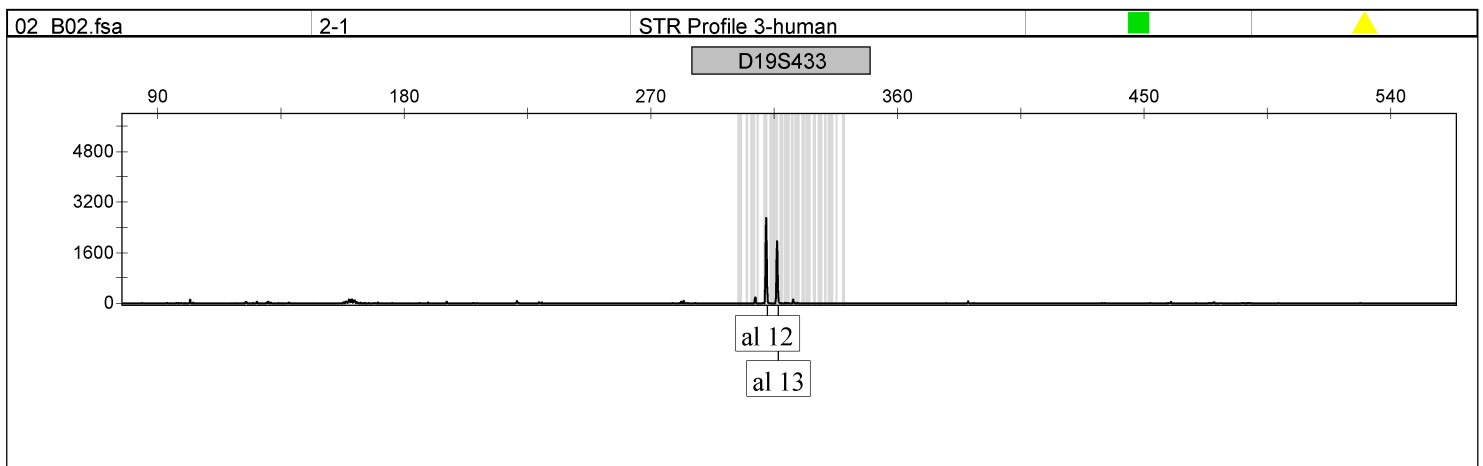

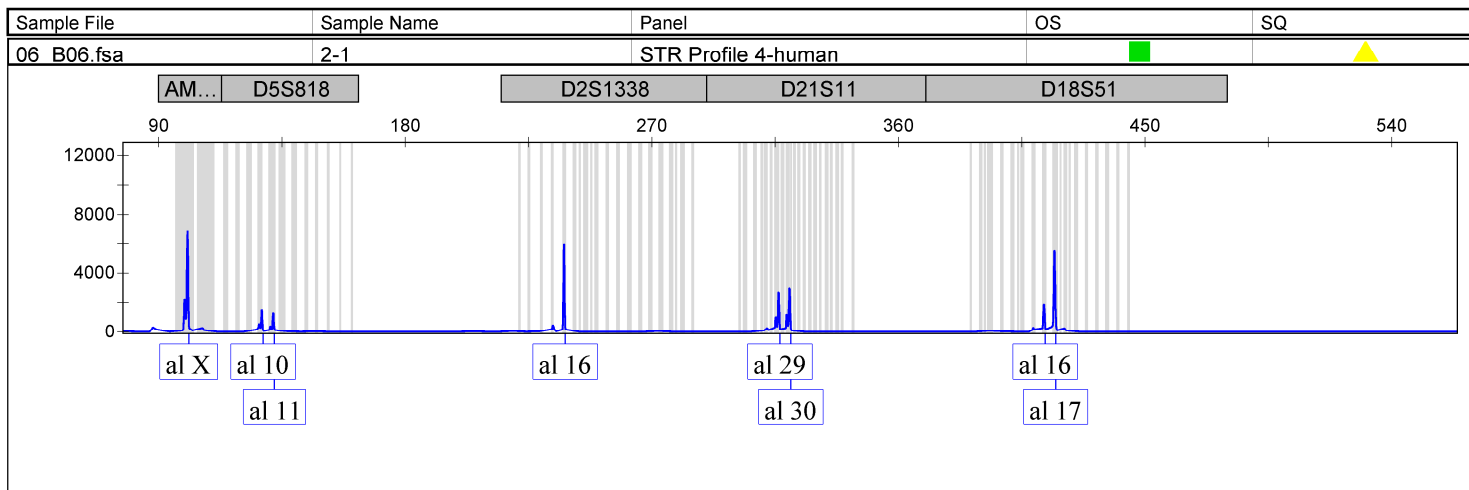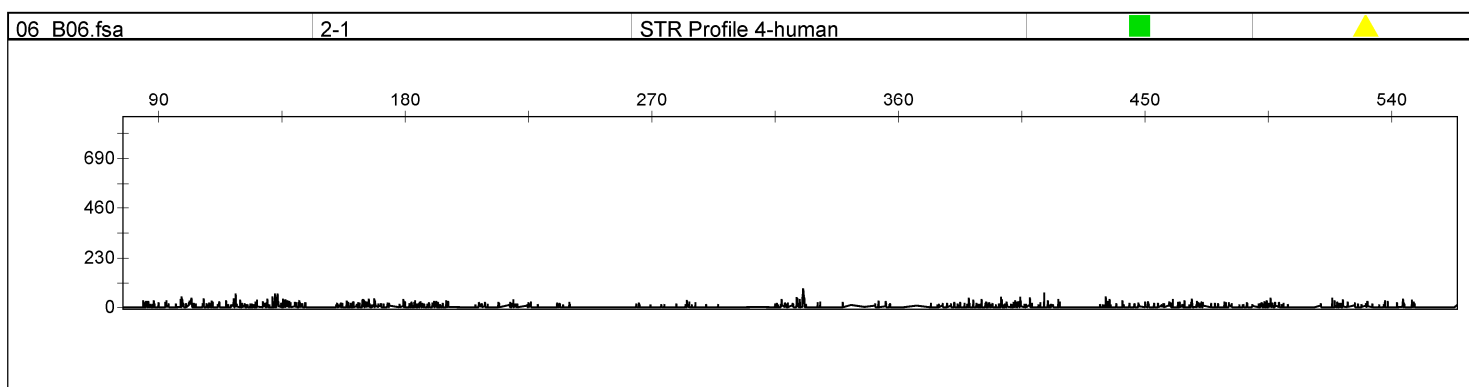

Supplement: Supplementary file 1 [file Data_Sheet_1.PDF]

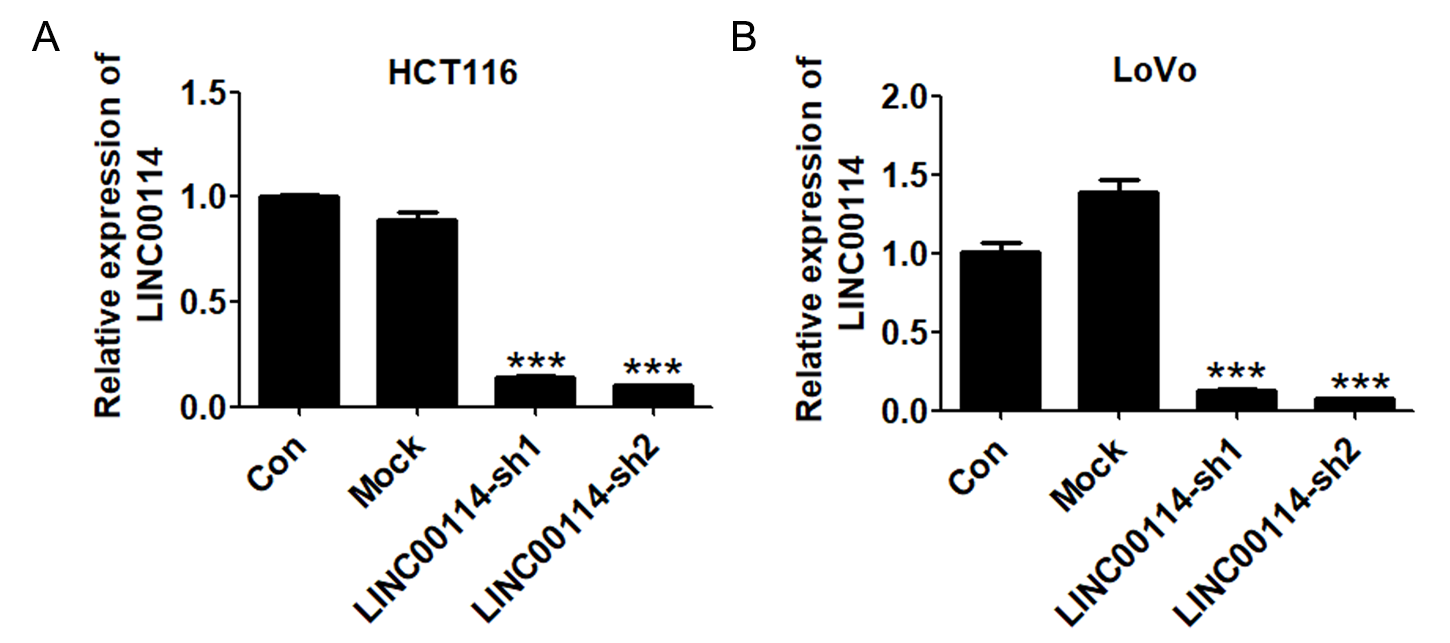

Supplement: Figure S1 — (A,B) Verification of the efficiency of LINC00114 interference plasmid in HCT116 and LoVo cells. ***P < 0.001. [file Image_1.TIF]
