# Supplementary material for: Long Non-coding RNA LINC00114 Facilitates Colorectal Cancer Development Through EZH2/DNMT1-Induced miR-133b Suppression
Source: Front Oncol. 2019 Dec 17;9:1383. doi: 10.3389/fonc.2019.01383 (PMC6928983; doi:10.3389/fonc.2019.01383)

GeneMapper 4.0

Cell Line Authentication 1229

Sample File	Sample Name	Panel	OS	SQ
09_B09.fsa	3-2	STR Profile 1-human		

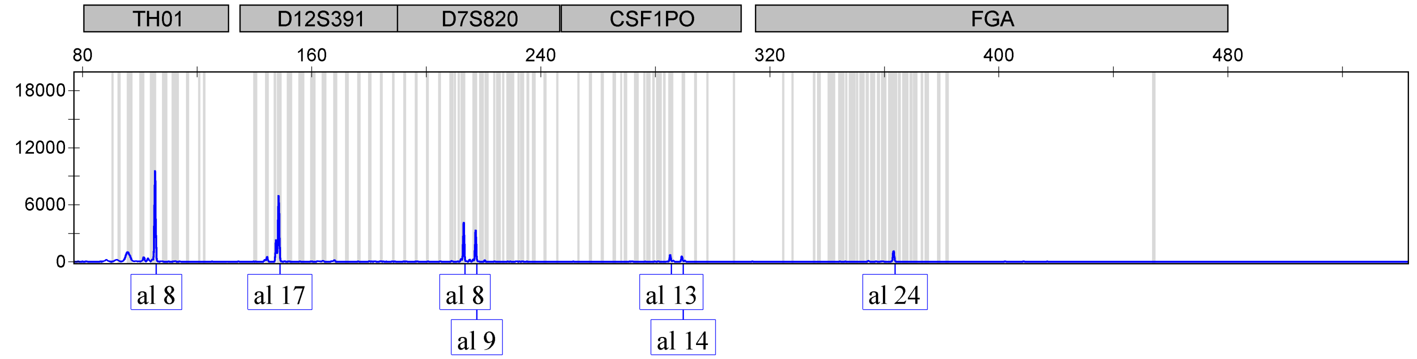

Sample File	Sample Name	Panel	OS	SQ
01_B01.fsa	3-2	STR Profile 2-human		

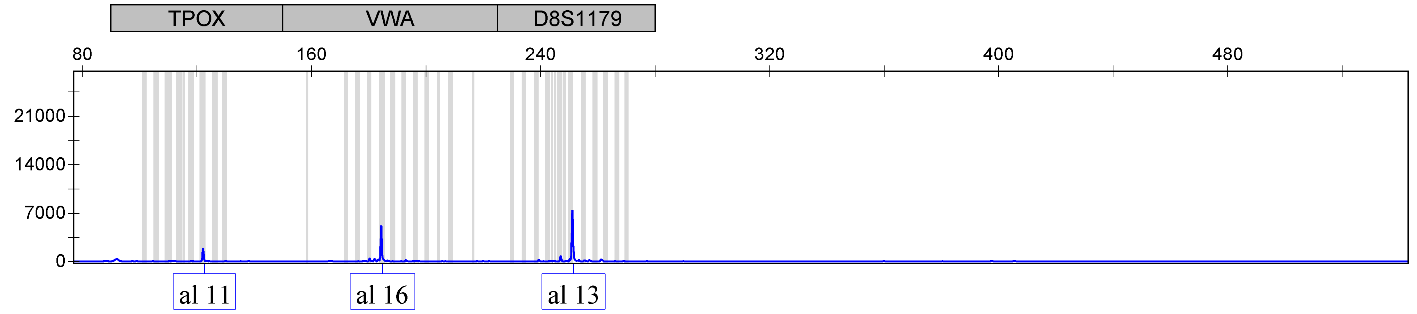

Sample File	Sample Name	Panel	OS	SQ
04_B04.fsa	3-2	STR Profile 3-human		

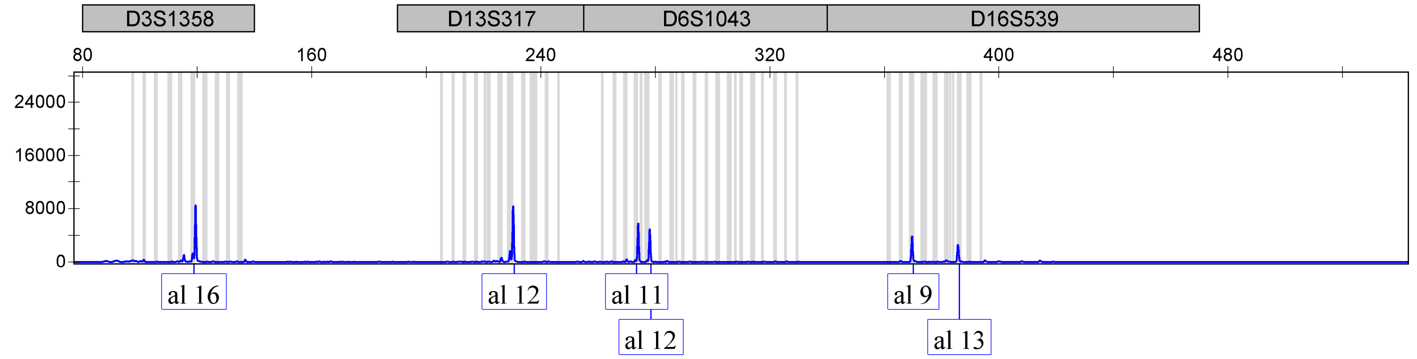

Sample File	Sample Name	Panel	OS	SQ
06_B06.fsa	3-2	STR Profile 4-human		

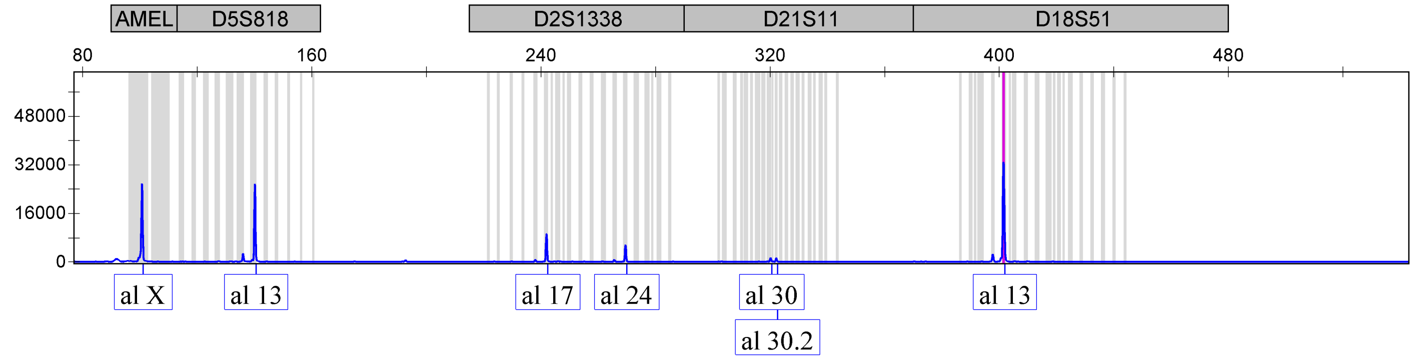

Cell Line Authentication 1229

Sample File	Sample Name	Panel	OS	SQ
01_B01.fsa	3-2	STR Profile 1-human		

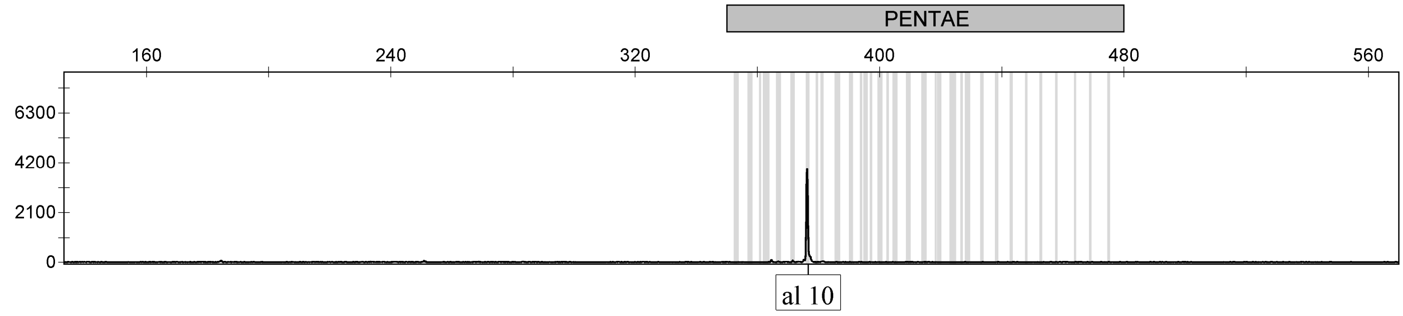

Sample File	Sample Name	Panel	OS	SQ
09_B09.fsa	3-2	STR Profile 2-human		

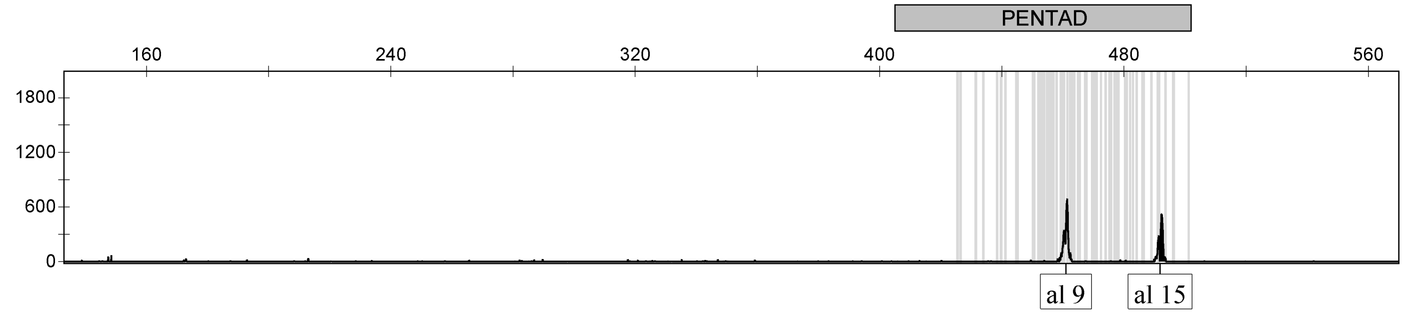

Sample File	Sample Name	Panel	OS	SQ
04_B04.fsa	3-2	STR Profile 3-human		

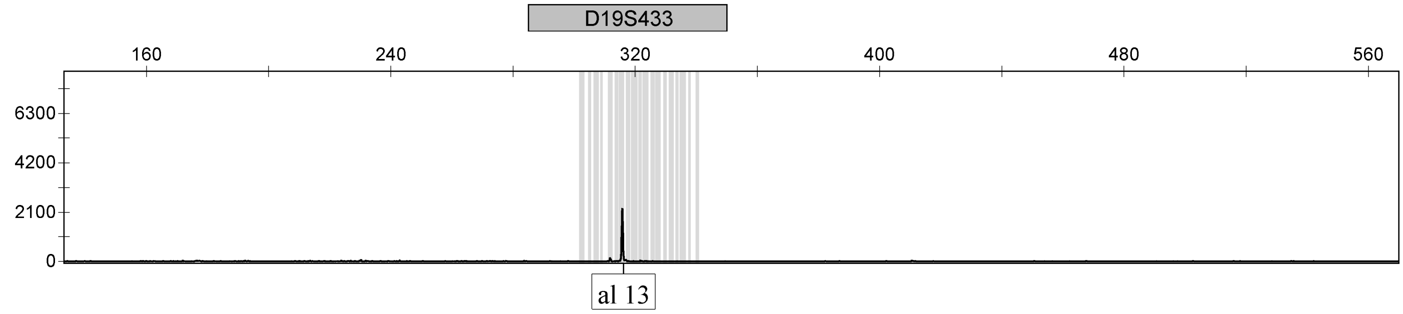

Supplement: Supplementary file 2 [file Data_Sheet_2.PDF]
