# Supplementary material for: Long Non-coding RNA LINC00114 Facilitates Colorectal Cancer Development Through EZH2/DNMT1-Induced miR-133b Suppression
Source: Front Oncol. 2019 Dec 17;9:1383. doi: 10.3389/fonc.2019.01383 (PMC6928983; doi:10.3389/fonc.2019.01383)

Applied Biosystems
GeneMapper 4.0

Cell Line Authentication 1213

Sample File	Sample Name	Panel	OS	SQ
01_D01.fsa	2-3	STR Profile 1-human		

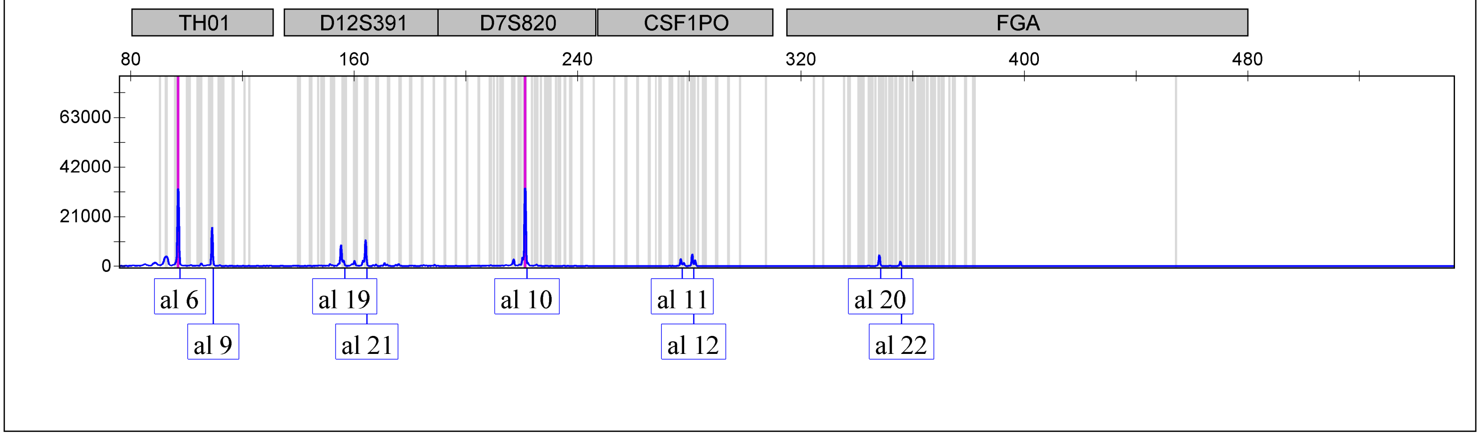

Sample File	Sample Name	Panel	OS	SQ
01_D01.fsa	2-3	STR Profile 1-human		

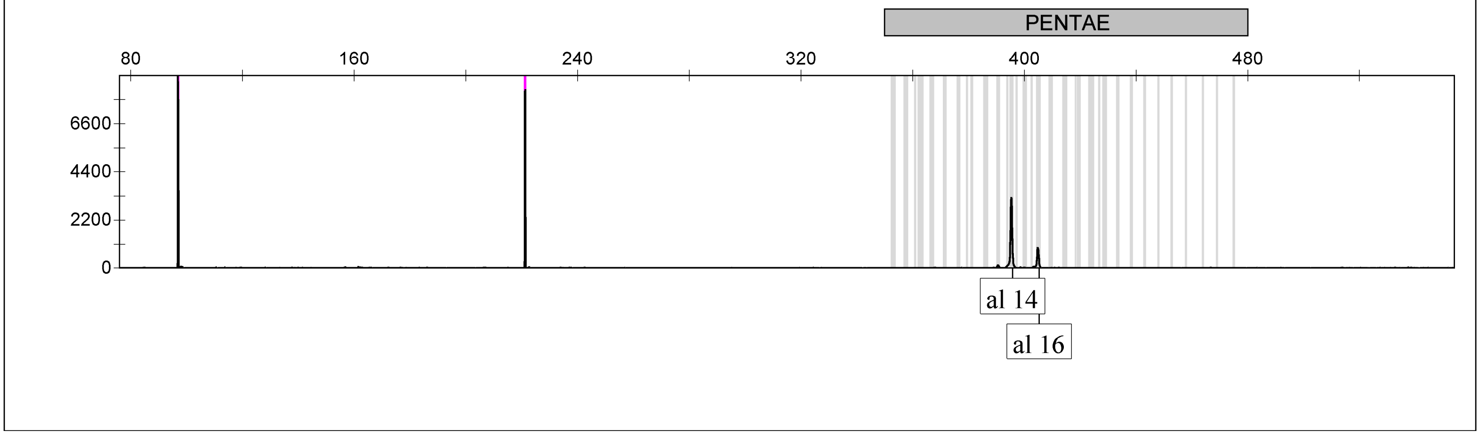

Sample File	Sample Name	Panel	OS	SQ
05_D05.fsa	2-3	STR Profile 2-human		

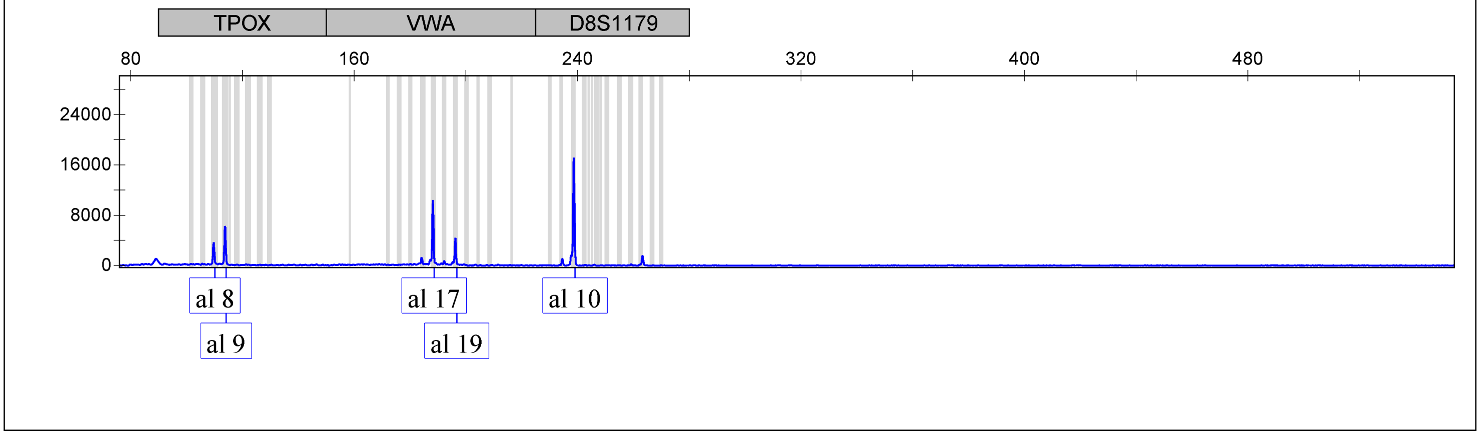

Sample File	Sample Name	Panel	OS	SQ
05 D05.fsa	2-3	STR Profile 2-human		

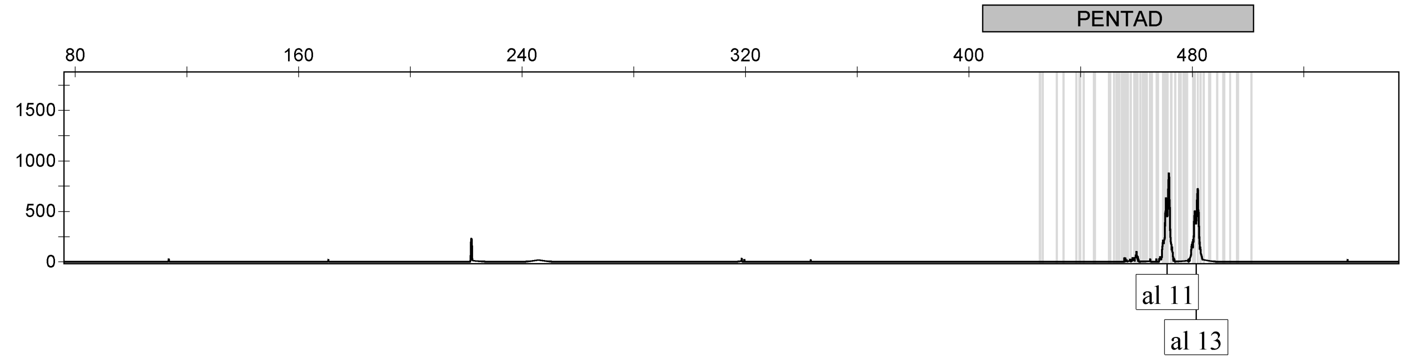

02 D02.fsa	2-3	STR Profile 3-human		

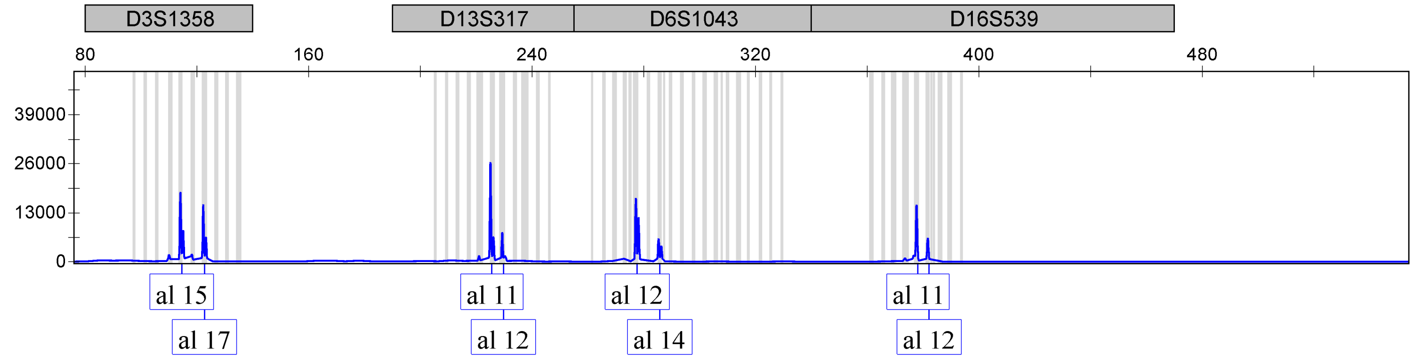

02 D02.fsa	2-3	STR Profile 3-human		

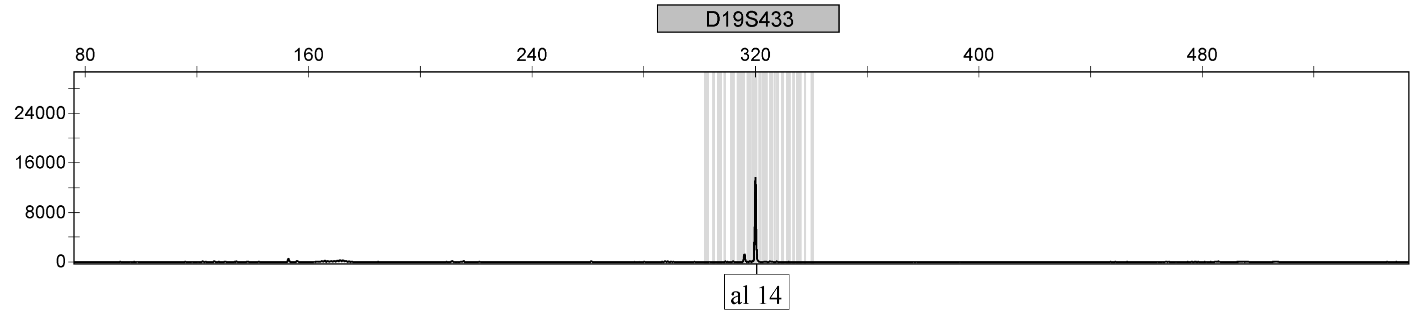

Applied Biosystems
GeneMapper 4.0

Cell Line Authentication 1213

Sample File	Sample Name	Panel	OS	SQ
06_D06.fsa	2-3	STR Profile 4-human		

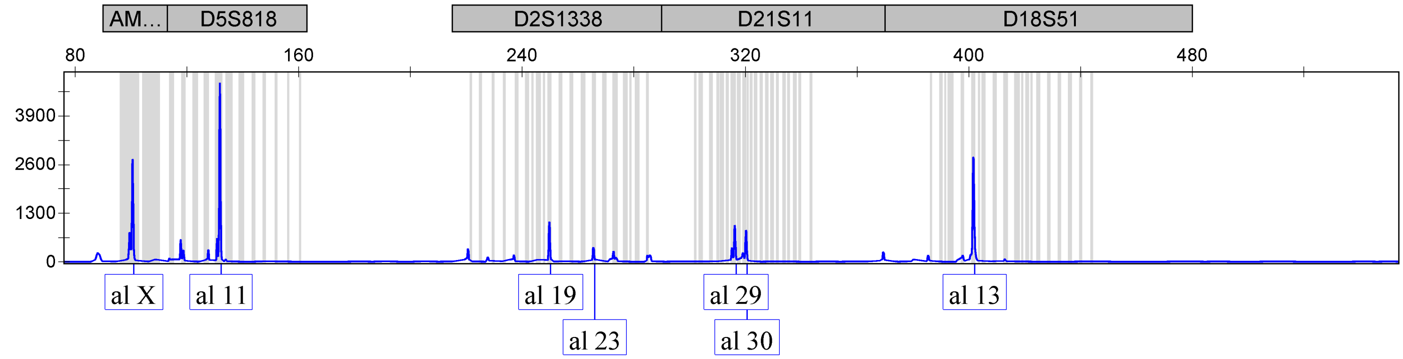

Sample File	Sample Name	Panel	OS	SQ
06_D06.fsa	2-3	STR Profile 4-human		

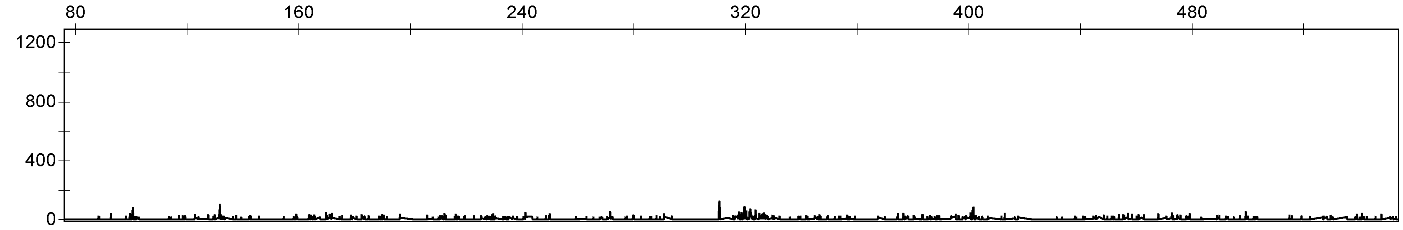

Supplement: Supplementary file 3 [file Data_Sheet_3.PDF]
