# Supplementary material for: Long Non-coding RNA LINC00114 Facilitates Colorectal Cancer Development Through EZH2/DNMT1-Induced miR-133b Suppression
Source: Front Oncol. 2019 Dec 17;9:1383. doi: 10.3389/fonc.2019.01383 (PMC6928983; doi:10.3389/fonc.2019.01383)
Supplement: Table S1 — Prediction of the binding ability of LINC00114 and mir-133b to genes. [file Table_1.docx]

| Table S1 Prediction of the binding ability of LINC00114 and mir-133b to genes | | |
| --- | --- | --- |
| Gene | Prediction method | Score |
| LINC00114 & EZH2 | Prediction using RF classifier | 0.8 |
|  | Prediction using SVM classifier | 0.96 |
| LINC00114 & DNMT1 | Prediction using RF classifier | 0.65 |
|  | Prediction using SVM classifier | 0.98 |
| LINC00114 & EZH1 | Prediction using RF classifier | 0.60 |
|  | Prediction using SVM classifier | 0.89 |
| LINC00114 & DNMT3b | Prediction using RF classifier | 0.55 |
|  | Prediction using SVM classifier | 0.98 |
| LINC00114 & EEZ | Prediction using RF classifier | 0.65 |
|  | Prediction using SVM classifier | 0.85 |
| LINC00114 & SUZ12 | Prediction using RF classifier | 0.75 |
|  | Prediction using SVM classifier | 0.88 |
| miR-133b & EZH2 | Prediction using RF classifier | 0.75 |
|  | Prediction using SVM classifier | 0.72 |
